# ﻿Taxonomic revision of the *Quasipaaverrucospinosa* complex (Amphibia, Dicroglossidae) in Vietnam, with descriptions of two new species

**DOI:** 10.3897/zookeys.1240.147337

**Published:** 2025-06-05

**Authors:** Cuong The Pham, Chung Van Hoang, Tien Quang Phan, Anh Van Pham, An Vinh Ong, Vien Hong Thi Nguyen, Thomas Ziegler, Truong Quang Nguyen

**Affiliations:** 1 Institute of Biology, Vietnam Academy of Science and Technology, 18 Hoang Quoc Viet Road, 10072 Hanoi, Vietnam; 2 Graduate University of Science and Technology, Vietnam Academy of Science and Technology, 18 Hoang Quoc Viet Road, 10072 Hanoi, Vietnam; 3 Faculty of Environmental Sciences, University of Science, Vietnam National University, Hanoi, 334 Nguyen Trai Road, 11400 Hanoi, Vietnam; 4 Department of Zoology, Vinh University, 182 Le Duan Road. Vinh City, Nghe An Province, Vietnam; 5 Faculty of Resources and Environment, Thai Nguyen University of Sciences, Thai Nguyen University, Tan Thinh Ward, Thai Nguyen City, Thai Nguyen 250000, Vietnam; 6 AG Zoologischer Garten Köln, Riehler Strasse 173, D-50735 Cologne, Germany; 7 Institute of Zoology, University of Cologne, Zülpicher Strasse 47b, D-50674 Cologne, Germany

**Keywords:** 16S rRNA, Cytb, molecular phylogeny, morphology, *Quasipaa*, taxonomy

## Abstract

This study provides a taxonomic revision of the *Quasipaaverrucospinosa* complex in Vietnam. Based on integrative taxonomic analyses, *Quasipaaverrucospinosa* sensu stricto is distributed in Lao Cai, Vinh Phuc, Ha Giang, and Tuyen Quang provinces. Other records of *Quasipaaverrucospinosa* in northern and central Vietnam revealed to be representatives of two new species: *Quasipaaohlerae***sp. nov.** from Son La, Thanh Hoa, and Nghe An provinces and *Quasipaabinhi***sp. nov.** from Quang Binh and Thua Thien Hue provinces. The two new species morphologically differ from each other and from other known species in the genus *Quasipaa* in size, skin texture, color pattern, and nuptial spines. The molecular analysis based on 16S rRNA and Cytb gene fragments showed that the genetic divergence between *Quasipaaohlerae***sp. nov.** and other congeners ranges from 2.96% (compared with *Q.delacouri* and *Quasipaabinhi***sp. nov.**) to 7.89% (compared with *Q.exilispinosa*) in the 16S gene and from 13.81% (compared with *Q.delacouri*) to 22.91% (compared with *Quasipaa* sp.) in the Cytb gene, while the p-distances between *Quasipaabinhi***sp. nov.** and its congeners ranges from 2.34% (compared with *Q.delacouri*) to 6.12% (compared with *Quasipaa* sp.) in the 16S gene and from 10.15% (compared with *Q.delacouri*) to 21.88% (compared with *Q.boulengeri*) in the Cytb gene. These new findings bring the total number of known species in the genus *Quasipaa* to 15 and the recorded species from Vietnam to eight.

## ﻿Introduction

The genus *Quasipaa* Dubois, 1992 is known from China throughout the Indochina region and southwards to Thailand (Frost 2024) and currently contains 13 recognized species (Frost 2024). In Vietnam, six species were recognized, viz. *Quasipaaacanthophora* Dubois & Ohler, 2009, *Q.boulengeri* (Günther, 1889), *Q.delacouri* (Angel, 1928), *Q.spinosa* (David, 1875), *Q.taoi* Pham, Hoang, Phan, Nguyen & Ziegler, 2022, and *Q.verrucospinosa* (Bourret, 1937). Recent phylogenetic studies showed that there are several unnamed lineages in the genus, indicating that its species richness remains underestimated (Che et al. 2009, 2010; Yan et al. 2021).

*Quasipaaverrucospinosa* (Bourret, 1937) was originally described based on the type series from Tam Dao (Vinh Phuc Province) and Sa Pa (Lao Cai Province). The species was subsequently reported from the North southwards to the Central Highlands of Vietnam (Nguyen et al. 2009; Frost 2024). Elsewhere, the species is known from Yunnan Province of China (Hu et al. 2005), as well as Xaisomboun, Phongsaly, and Xekong provinces of Laos (Nguyen et al. 2020), and Doi Phu Kha National Park, Nan Province of Thailand (Suwannapoom et al. 2021). This is a cryptic species and has genetic differences amongst populations. Therefore, a comprehensive assessment of the taxonomic status of this species should be prioritized and a degree of urgency should be applied to determine the true distribution area of this species (Suwannapoom et al. 2021).

During our field surveys between 2012 and 2025, a new series of *Quasipaa* was collected in northern and central Vietnam and they were placed to the Group A in Suwannapoom et al. (2021) based on molecular data. Closer morphological examination showed that *Q.verrucospinosa* sensu stricto has a restricted distribution in northeastern Vietnam, whereas the populations from northwestern and Central Vietnam reveal two unnamed taxa. They could be clearly distinguished from other known species of *Quasipaa* in size, skin texture, color pattern, and nuptial spines. In the phylogenetic analyses, these taxa were clearly separated from each other and from their congeners with a genetic divergence ranging from 2.34% to 8.60% in the 16S gene fragment and from 10.15% to 22.91% in the Cytb gene fragment. Therefore, we herein describe the unnamed taxa as two new species.

## ﻿Materials and methods

### ﻿Sampling

Field surveys were conducted in August 2012, in October and November 2021 in Xuan Lien Nature Reserve, Thuong Xuan District, Thanh Hoa Province; in December 2012, in September 2014 and in September 2016 in Copia Nature Reserve, Son La Province; in April and October 2015 in Le Thuy District, Quang Binh Province; in November 2015 in Bac Me Nature Reserve, Ha Giang Province; in April 2017 in A Luoi District, Thua Thien-Hue Province; in August 2017 in Na Hang District, Tuyen Quang Province; in October 2018 in Cham Chu Nature Reserve, Ham Yen District, Tuyen Quang Province; in October 2021 and April 2025 in Pu Hoat Nature Reserve, Que Phong District, Nghe An Province; in July 2022 in Tam Dao National Park, Vinh Phuc Province; in October 2022 in Hoang Lien National Park, Sa Pa District, Lao Cai Province (the type locality of *Quasipaaverrucospinosa*).

Frogs were collected by hand between 19:00 and 23:00 following the guidelines approved by the American Society of Ichthyologists and Herpetologists for animal care (Beaupre et al. 2004). After taking photographs in life, frogs were anaesthetized and euthanized in a closed vessel with a piece of cotton wool containing ethyl acetate (Simmons 2002), fixed in 80% ethanol for five hours, and later transferred to 70% ethanol for permanent storage. Tissue samples were preserved separately in 70% ethanol prior to fixation. Voucher specimens referred to in this paper were deposited in the collections of the Institute of Biology (**IB**) (formerly known as the Institute of Ecology and Biological Resources, **IEBR**), Hanoi, Vietnam and the Zoological Museum, Vietnam National University, Hanoi (**ZVNU**), Vietnam.

### ﻿Molecular data and phylogenetic analyses

In this study, tissue samples were extracted using PureLink™ RNA Micro Scale Kit (Thermo Fisher Scientific company), following the manufacturer’s instructions. DNA was amplified using PCR Applied Biosystems. PCR volume consisted of 25 μl, including 12 μl of Mastermix, 6 μl of water, 1 μl of each primer at concentration of 10 pmol/μl, and 5 μl of DNA. A total of 20 samples of seven species of *Quasipaa* and one samples of *Nanoranayunnanensis* (Outgroup) were used for molecular analysis with 16S (~ 590 base pairs) mitochondrial gene fragment (Table 1). PCR conditions: 94 °C for 5 min of initial denaturation; with 35 cycles of denaturation at 94 °C for 30 s, annealing at 56 °C for 30 s, and extension at 72 °C for 45 s; and the final extension at 72 °C for 7 min. Additionally, 15 samples of eight species of *Quasipaa* were used for molecular analysis with Cytb (~ 630 base pairs) mitochondrial gene fragment (Table 1). PCR conditions: 94 °C for 5 min of initial denaturation; with 35 cycles of denaturation at 94 °C for 1 min, annealing at 45 °C for 45 s, and extension at 72 °C for 1 min; and the final extension at 72 °C for 10 min. PCR products were sent to Apical Scientific Company (Malaysia) for sequencing (https://apicalscientific.com).

**Table 1.** GenBank accession numbers and associated samples used in this study.

	Species	Location	Voucher	GenBank Acc. Number		Reference
				16S	Cytb	
1	*Quasipaaohlerae* sp. nov.	Nghe An, Vietnam	IEBR A.5167	PV475529	PV478085	This study
2	*Quasipaaohlerae* sp. nov.	Thanh Hoa, Vietnam	IEBR A.5159	PV475528	PV478082	This study
3	*Quasipaaohlerae* sp. nov.	Thanh Hoa, Vietnam	IEBR A.5160	PV475527	PV478083	This study
4	*Quasipaaohlerae* sp. nov.	Thanh Hoa, Vietnam	IEBR A.5161	PV475526	PV478084	This study
5	*Quasipaaohlerae* sp. nov.	Thanh Hoa, Vietnam	IEBR A.5164	PV475525		This study
8	*Quasipaaohlerae* sp. nov.	Nghe An, Vietnam	FMNH255623	EU979810		Che et al. (2009)
12	*Quasipaabinhi* sp. nov.	Quang Binh, Vietnam	IEBR A.5174	PV475530		This study
13	*Quasipaabinhi* sp. nov.	Quang Binh, Vietnam	IEBR A.5175	PV475531	PV478086	This study
14	*Quasipaabinhi* sp. nov.	Da Nang, Vietnam	KIZ013695		MT678119	Yan et al. (2021)
15	*Quasipaabinhi* sp. nov.	Da Nang, Vietnam	KIZ013702		MT678120	Yan et al. (2021)
16	*Quasipaabinhi* sp. nov.	Thua Thien Hue, Vietnam	KIZ010072		MT678113	Yan et al. (2021)
17	*Quasipaa* sp.	Phu Tho, Vietnam	IEBR A.6362	PV475532		This study
18	*Quasipaa* sp.	Phu Tho, Vietnam	IEBR A.6363	PV475533		This study
19	*Quasipaa* sp.	Phu Tho, Vietnam	IEBR A.6364	PV475534		This study
20	*Quasipaa* sp.	Phu Tho, Vietnam	IEBR A.5171	PV475535	PV478087	This study
21	*Quasipaa* sp.	Yunnan, China	KizYP008	DQ118480		Che et al. (2009)
22	*Quasipaa* sp.	Yunnan, China	KIZYP007	DQ118481	MT678239	Che et al. (2009), Yan et al. (2021)
23	*Quasipaa* sp.	Yunnan, China	KIZYN080408		MT678240	Yan et al. (2021)
24	*Q.delacouri*	Tuyen Quang, Vietnam	IEBR A.5168	OP326696		Pham et al. (2022)
25	*Q.delacouri*	Tuyen Quang, Vietnam	IEBR A.5169	OP326697		Pham et al. (2022)
26	*Q.delacouri*	Tuyen Quang, Vietnam	IEBR A.5017	OP326698		Pham et al. (2022)
27	*Q.delacouri*	Phu Tho, Vietnam	IEBR A.5020	PV475536	PV478088	This study
28	*Q.taoi*	Kon Tum, Vietnam	IEBR A.4997	OP326684	PV478089	Pham et al. (2022), This study
29	*Q.taoi*	Kon Tum, Vietnam	IEBR A.4998	OP326685	PV478090	Pham et al. (2022), This study
30	*Q.taoi*	Kon Tum, Vietnam	ROM37390	EU979804		Che et al. (2009)
31	*Q.taoi*	Kon Tum, Vietnam	VNMN1604		MT678257	Yan et al. (2021)
32	*Q.taoi*	Xekong, Laos	FMNH258383	EU979803		Che et al. (2009)
33	*Q.verrucospinosa*	Vinh Phuc, Vietnam (type locality)	MVZ223858	EU979813		Che et al. (2009)
34	*Q.verrucospinosa*	Vinh Phuc, Vietnam (type locality)	IEBR A.5155	PV475537	PV478091	This study
35	*Q.verrucospinosa*	Tuyen Quang, Vietnam	IEBR A.5025	OP326686		Pham et al. (2022)
36	*Q.verrucospinosa*	Tuyen Quang, Vietnam	IEBR A.5026	OP326687		Pham et al. (2022)
37	*Q.verrucospinosa*	Tuyen Quang, Vietnam	IEBR A.5027	OP326688		Pham et al. (2022)
38	*Q.verrucospinosa*	Tuyen Quang, Vietnam	IEBR A.5028	PV475538	PV478092	This study
39	*Q.verrucospinosa*	Ha Giang, Vietnam	IEBR A.5022	PV475539	PV478093	This study
40	*Q.verrucospinosa*	Bac Kan, Vietnam	IEBR A.5176	PV475540		This study
41	*Q.verrucospinosa*	Lao Cai, Vietnam (type locality)	IEBR A.5172	PV475541		This study
42	*Q.verrucospinosa*	Lao Cai, Vietnam (type locality)	IEBR A.5173	PV475542		This study
43	*Q.yei*	Henan, China	KIZYP155	DQ118488		Che et al. (2009)
44	*Q.yei*	Henan, China	KIZ02412		MT678134	Yan et al. (2021)
45	*Q.shini*	Guangxi, China	KIZYP012	DQ118487		Che et al. (2009)
46	*Q.shini*	Guangxi, China	KIZ021613		MT678128	Yan et al. (2021)
47	*Q.boulengeri*	Cao Bang, Vietnam	IEBR A.5007	OP326690		Pham et al. (2022)
48	*Q.boulengeri*	Cao Bang, Vietnam	IEBR A.5008	OP326691		Pham et al. (2022)
49	*Q.boulengeri*	Cao Bang, Vietnam	IEBR A.5039	OP326692		Pham et al. (2022)
50	*Q.boulengeri*	Cao Bang, Vietnam	IEBR A.5040	OP326693		Pham et al. (2022)
51	*Q.boulengeri*	Cao Bang, Vietnam	IEBR A.5041	PV475543	PV478094	This study
52	*Q.boulengeri*	Ha Giang, Vietnam	IEBR A.5015	PV475544	PV478095	This study
53	*Q.boulengeri*	Hebei, China	KIZ-HUB292	EU979815		Che et al. (2009)
54	*Q.boulengeri*	Hunan, China	KIZ04604		MT678158	Yan et al. (2021)
55	*Q.boulengeri*	Hunan, China	KIZ04605		MT678159	Yan et al. (2021)
56	*Q.boulengeri*	Hunan, China	KIZ04609		MT678160	Yan et al. (2021)
57	*Q.boulengeri*	Guizhou, China	KIZ048646		MT678163	Yan et al. (2021)
58	*Q.boulengeri*	Guizhou, China	KIZ048647		MT678164	Yan et al. (2021)
59	*Q.boulengeri*	Guangxi, China	KIZ08459		MT678184	Yan et al. (2021)
61	Q.cf.boulengeri	Guizhou, China	KIZ07984		MT678182	Yan et al. (2021)
62	*Q.exilispinosa*	Fujian, China	KIPYP020	DQ118484		Che et al. (2009)
63	*Q.exilispinosa*	Fujian, China	KIZ08933		MT678197	Yan et al. (2021)
64	*Q.exilispinosa*	Fujian, China	KIZ08934		MT678198	Yan et al. (2021)
65	*Q.exilispinosa*	Fujian, China	KIZ08935		MT678199	Yan et al. (2021)
66	*Q.exilispinosa*	Fujian, China	KIZ08936		MT678200	Yan et al. (2021)
67	*Q.exilispinosa*	Fujian, China	KIZ08937		MT678201	Yan et al. (2021)
68	*Q.exilispinosa*	Fujian, China	KIZ08938		MT678202	Yan et al. (2021)
69	*Q.exilispinosa*	Fujian, China	KIZ08939		MT678203	Yan et al. (2021)
70	*Q.spinosa*	Zhejiang, China	Sample No. 003	FJ432700	FJ432700	Zhou et al. (2009)
71	*Q.spinosa*	Zhejiang, China	KIZ03486		MT678143	Yan et al. (2021)
72	*Q.acanthophora*	Lang Son, Vietnam	IEBR A.5030	OP326694	PV478096	Pham et al. (2022), This study
73	*Q.acanthophora*	Lang Son, Vietnam	IEBR A.5031	OP326695		Pham et al. (2022)
74	*Q.acanthophora*	Guangxi, China	KIZ09282		MT678226	Yan et al. (2021)
75	*Q.acanthophora*	Guangxi, China	KIZ09283		MT678227	Yan et al. (2021)
76	*Q.acanthophora*	Guangxi, China	KIZYPX26579		MT678248	Yan et al. (2021)
77	*Q.acanthophora*	Guangxi, China	KIZYPX26580		MT678249	Yan et al. (2021)
78	*Q.acanthophora*	Guangxi, China	KIZ021611		MT678127	Yan et al. (2021)
79	*Q.acanthophora*	Guangxi, China	KIZ022191		MT678129	Yan et al. (2021)
80	*Q.acanthophora*	Guangxi, China	KIZ022192		MT678130	Yan et al. (2021)
81	*Q.jiulongensis*	Fujian, China		KF199149		Zhang et al. (2018)
82	*Q.jiulongensis*	Fujian, China	KIZ08918		MT678192	Yan et al. (2021)
83	*Q.jiulongensis*	Fujian, China	KIZ08919		MT678193	Yan et al. (2021)
**Out group**						
	*Nanoranaparkeri*	Yunnan, China	NC_026789	KP317482	KP317483	Jiang et al. (2016)
	*Nanoranaarnoldi*	Yunnan, China	SCUM050410CHX	EU979838		Che et al. (2009)
	*Nanoranayunnanensis*	Son La, Vietnam	IEBR A.5177	PV475545		This study

In addition, we used 29 available sequences of 16S rRNA of the genus *Quasipaa* in GenBank for phylogenetic analyses (Che et al. 2009; Zhou et al. 2009; Zhang et al. 2018; Yan et al. 2021; Pham et al. 2022). Two sequences of *Nanoranaparkeri* and *N.arnoldi* were included in the analysis as outgroup (Che et al. 2009; Jiang et al. 2016). We used 36 available sequences of Cytb rRNA of the genus *Quasipaa* in GenBank for phylogenetic analyses (Che et al. 2009; Zhou et al. 2009; Yan et al. 2021; Pham et al. 2022). A sequence of *N.parkeri* was included in the analysis as the outgroup (Che et al. 2009; Jiang et al. 2016). For locality information and accession numbers for all sequences used in this study see Table 1.

Phylogenetic trees were constructed by using maximum likelihood (ML) and Bayesian inference (BI). Chromas Pro software (Technelysium Pty Ltd, Tewantin, Australia) was used to edit the sequences, which were aligned using the ClustalW (Thompson et al. 1997) option in MEGA11 (Tamura et al. 2021) with default parameters and subsequently optimized manually in BioEdit v. 7.0.5.2 (Hall 1999). We then checked the initial alignments by eye and adjusted slightly. Evolutionary analyses were conducted in MEGA11 (Tamura et al. 2021). Prior to ML and Bayesian phylogenetic analyses, we chose the optimum substitution models for entire sequences using Kakusan 4 (Tanabe 2011) based on the Akaike information criterion (AIC). The BI was performed in MrBayes v. 3.2.7a (Tanabe 2007). The optimal model for BI analysis was GTR Gamma. The BI summarized two independent runs of four Markov Chains for 10 million generations. A tree was sampled every 100 generations and a consensus topology was calculated after discarding the first 25% of trees (Nguyen et al. 2017). We checked parameter estimates and convergence using Tracer v. 1.7.1 (Rambaut et al. 2018). The strength of nodal support in the ML tree was analyzed using non-parametric bootstrapping with 1000 replicates. We regarded tree nodes in the ML tree with bootstrap values of 75% or greater as sufficiently resolved (Hillis and Bull 1993; Huelsenbeck and Hillis 1993), and nodes with a BPP of 95% or greater as significant in the BI analysis (Leaché and Reeder 2002).

### ﻿Morphological analysis

Measurements were taken with digital calipers to the nearest 0.1 mm. The following abbreviations are used (Pham et al. 2022):

**SVL** snout-vent length (from tip of snout to cloaca);

**HL** head length (a parallel line with the vertebral column from posterior margin of mandible to tip of snout);

**HW** maximum head width (at rictus);

**RL** rostral length (from anterior corner of orbit to tip of snout);

**NS** distance from nostril to tip of snout;

**EN** distance from anterior corner of orbit to nostril;

**IND** internarial distance (distance between nostrils);

**IOD** interorbital distance;

**ED** eye diameter;

**UEW** maximum width of upper eyelid;

**DAE** distance between anterior margins of orbits;

**DPE** distance between posterior margins of orbits;

**MN** distance from posterior margin of mandible to nostril;

**MFE** distance from posterior margin of mandible to anterior margin of orbit;

**MBE** distance from posterior margin of mandible to posterior margin of eye;

**TD** tympanum diameter;

**TYE** distance from anterior margin of tympanum to posterior corner of orbit;

**UAL** upper arm length (from axilla to elbow);

**FAL** fore arm length (from elbow to tip of third finger);

**FL1–4** finger length I–IV (from inner to outer);

**NPL** nuptial pad length - finger I;

**FeL** femur length (from vent to knee);

**TbL** tibia length (from knee to tarsus);

**TbW** maximum tibia width;

**FoL** foot length (from tarsus to tip of fourth toe);

**TL1–5** toe length I–V;

**IMT** inner metatarsal tubercle length.

For webbing formula, we followed Glaw and Vences (2007). Sex was determined by gonadal inspection.

Morphological comparisons were based on specimens examination (Appendix 1) and data from literature (e.g., Angel 1928; Bourret 1937, 1942; Liu 1950; Inger 1970; Liu and Hu 1975; Huang and Liu 1985; Wu and Zhao 1995; Inger et al. 1999; Chen et al. 2002; Ohler and Dubois 2006; Dubois and Ohler 2009; Fei et al. 2009, 2012; Pham et al. 2022).

### ﻿Principal component analysis (PCA)

For morphometric comparisons, we used 13 specimens (seven males and six females) of *Quasipaaohlerae* sp. nov. from Thanh Hoa, Nghe An and Son La provinces; nine specimens (five males and four females) of *Quasipaabinhi* sp. nov. from Quang Binh and Thua Thien-Hue provinces; 14 specimens (seven males and seven females) of *Quasipaaverrucospinosa* sensu stricto from Vinh Phuc and Lao Cai provinces; and six specimens (three males and three females) of *Q.delacouri* from Ha Giang, Tuyen Quang, and Phu Tho provinces. All statistical analyses were performed using PAST v. 4.11 software (Hammer et al. 2001).

## ﻿Results

### ﻿Phylogenetic analyses

**Figure 1.**
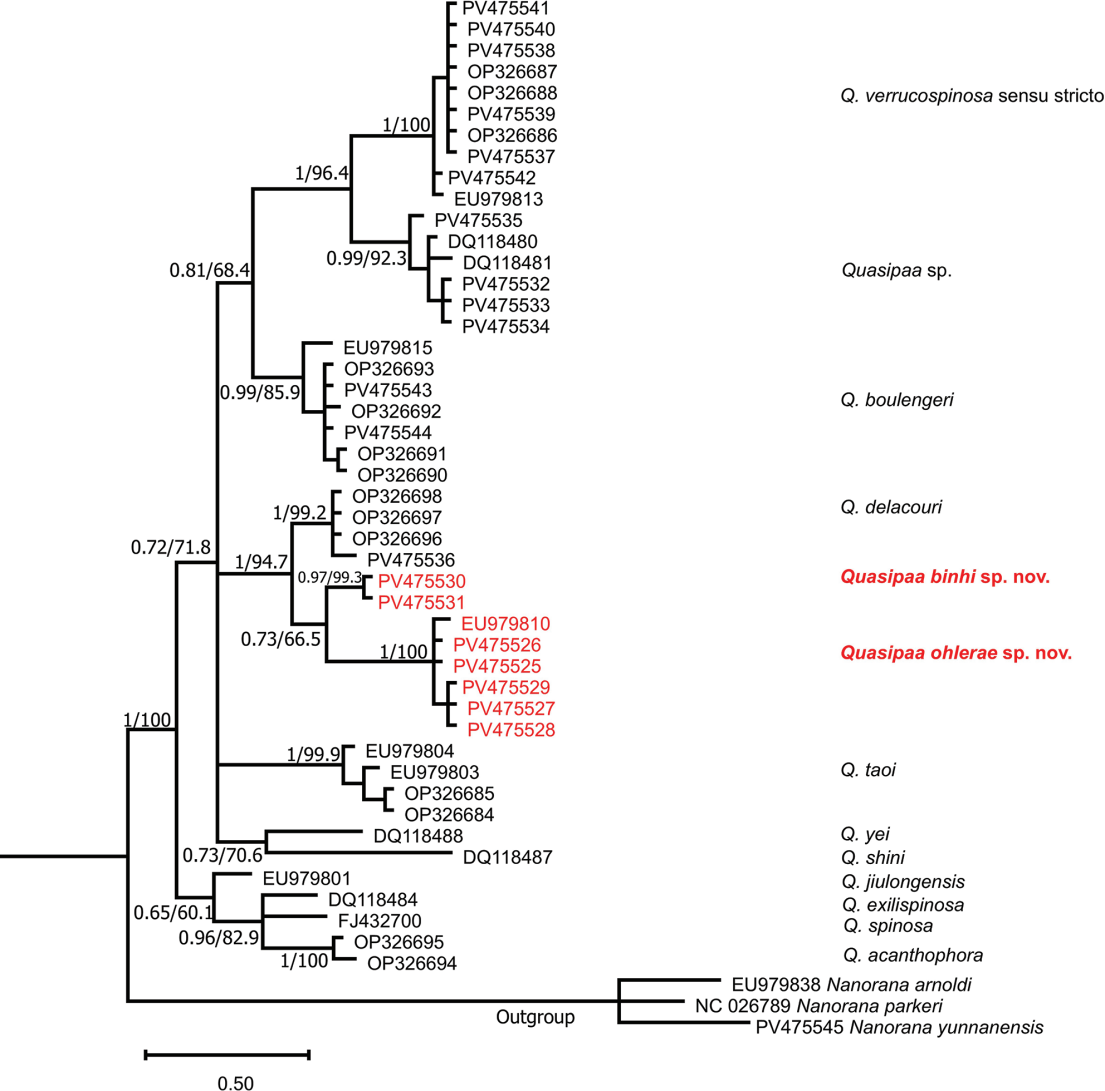
Bayesian phylogram based on a partial 16S mitochondrial fragment. Numbers above and below branches are MP/ML bootstrap values and Bayesian posterior probabilities (> 50%), respectively. Hyphen denotes < 50% value. Bold text highlights new samples collected within this study.

16S rRNA gene: The combined matrix contained 527 aligned characters. Of those, 425 sites were conserved, and 102 sites were variable, of which 80 were found to be potentially parsimony informative. The estimated Transition/Transversion bias (R) is 3.374. Substitution pattern and rates were estimated under the Tamura (1992) model. The nucleotide frequencies are A = 30.94%, T/U = 23.29%, C = 24.64%, and G = 21.13%. In terms of pairwise genetic distance, interspecific uncorrected *p*-distance of the *Quasipaa* species ranged from 2.32–2.92% (between *Q.acanthophora* and *Q.jiulongensis*) to 8.71% (between *Q.shini* and *Q.exilispinosa*) (Table 2). The genetic divergence between *Quasipaaohlerae* sp. nov. and its congeners ranged from 2.96–3.56% (*Quasipaabinhi* sp. nov.) to 7.27–7.89% (*Q.exilispinosa*), which was higher or similar to that between *Q.acanthophora* and *Q.jiulongensis* (2.32–2.92%); between *Quasipaabinhi* sp. nov. and *Q.delacouri* (2.34–2.93%); between *Q.exilispinosa* and *Q.acanthophora* (3.54–4.15%); between *Q.exilispinosa* and *Q.spinosa* (2.94%); between *Q.exilispinosa* and *Q.jiulongensis* (3.52%); between *Q.jiulongensis* and *Q.spinosa* (2.91%); between *Q.acanthophora* and *Q.spinosa* (2.92–3.53%); and between *Q.jiulongensis* and *Q.boulengeri* (2.33–4.14%) (Table 2). The genetic divergence of *Quasipaabinhi* sp. nov. and its congeners ranged from 2.34–2.93% (*Q.delacouri*) to 6.12% (*Quasipaa* sp.). The ML and BI analyses produced topologies with –ln L = 1928.023 and 2030.328, respectively, with a gamma shape parameter (G: 0.105 in ML and 0.13 in BI). Phylogenetic analyses employing ML and BI methods were nearly identical, with most well-supported nodes on the ML tree also well-supported on the BI tree, and only the BI tree is presented in Fig. 1.

**Table 2.** Uncorrected *p*-distance matrix showing percentage pairwise genetic divergences (%) for the 16S rRNA gene between members of the genus *Quasipaa*.

		1	2	3	4	5	6	7	8	9	10	11	12	13
1	*Quasipaaohlerae* sp. nov.	**0.00–0.58**												
2	*Quasipaabinhi* sp. nov.	2.96–3.56	**0.00**											
3	*Q.delacouri*	2.96–4.18	2.34–2.93	**0.00–0.58**										
4	*Q.verrucospinosa*	6.67–7.29	6.08	5.43–6.06	**0.00**									
5	*Q.boulengeri*	4.19–6.03	4.21–4.83	3.58–4.81	4.76–5.98	**0.00–1.74**								
6	*Quasipaa* sp.	6.65–8.60	4.83–6.12	5.41–7.36	3.51–4.74	4.14–4.74	**0.00–1.75**							
7	*Q.taoi*	4.76–5.98	4.79–5.40	4.15–4.77	5.38	4.76–5.37	5.37–6.65	**0.00–0.57**						
8	*Q.acanthophora*	4.74–5.97	3.56–4.18	2.35–3.56	7.25–7.89	3.54–6.01	5.99–7.94	5.33–6.57	**0.00–0.57**					
9	*Q.spinosa*	5.35–5.96	4.16	2.94–3.55	6.59	4.14–4.74	5.35–6.63	4.70–5.30	2.92–3.53	**0.00**				
10	*Q.jiulongensis*	4.74–5.35	3.56	3.54–4.15	6.59	2.33–4.14	5.35–6.63	4.10–4.70	2.32–2.92	2.91	**0.00**			
11	*Q.yei*	3.52–4.12	4.15	2.93–3.54	7.87	5.38–5.99	7.85–9.19	5.33–5.94	4.71–5.33	5.32	4.71	**0.00**		
12	*Q.shini*	4.86	5.50	4.23–4.86	7.36	4.84–5.46	7.34–8.69	6.10	6.08–6.73	6.71	6.08	5.45	**0.00**	
13	*Q.exilispinosa*	7.27–7.89	6.06	4.80–5.43	8.55	4.77–5.38	6.65–7.97	6.59–7.21	3.54–4.15	2.94	3.52	7.22	8.71	**0.00**

**Figure 2.**
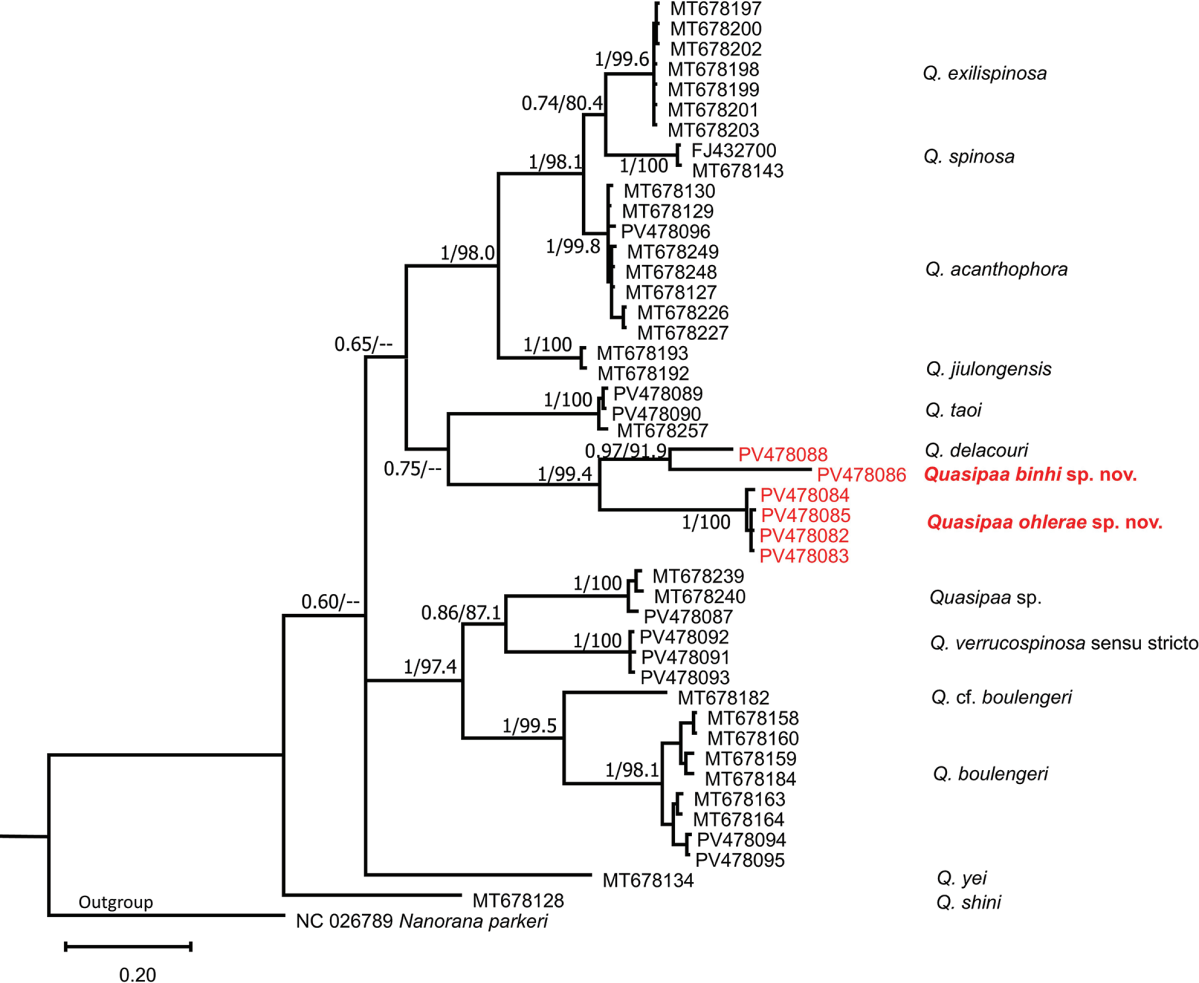
Bayesian phylogram based on a partial Cytb mitochondrial fragment. Numbers above and below branches are MP/ML bootstrap values and Bayesian posterior probabilities (> 50%), respectively. Hyphen denotes < 50% value. Bold text highlights new samples collected within this study.

Cytb rRNA gene: The combined matrix contained 607 aligned characters. Of those, 364 sites were conserved, and 243 sites were variable, of which 212 were found to be potentially parsimony informative. The estimated Transition/Transversion bias (R) is 2.87. Substitution pattern and rates were estimated under the Tamura (1992) model. The nucleotide frequencies are A = 24.60%, T/U = 28.93%, C = 31.83%, and G = 15.09%. In terms of pairwise genetic distance, interspecific uncorrected *p*-distance of the *Quasipaa* species ranged from 6.20–6.75% (between *Q.exilispinosa* and *Q.acanthophora*) to 22.20–22.91% (between *Quasipaaohlerae* sp. nov. and *Quasipaa* sp.) (Table 3). The genetic divergence of *Quasipaaohlerae* sp. nov. and its congeners ranged from 13.81–14.21% (*Q.delacouri*) to 22.20–22.91% (*Quasipaa* sp.), which was greater than that between *Q.exilispinosa* and *Q.acanthophora* (6.20–6.75%); between *Q.acanthophora* and *Q.spinosa* (7.82–8.93%); between *Q.exilispinosa* and *Q.spinosa* (7.65–8.02%); between *Q.jiulongensis* and *Q.acanthophora* (10.94–11.54%); between *Q.exilispinosa* and *Q.jiulongensis* (12.70–13.09%); and between *Q.jiulongensis* and *Q.spinosa* (12.26–12.6%). The genetic divergence of *Quasipaabinhi* sp. nov. and its congeners ranged from 10.15% (*Q.delacouri*) to 21.35–21.66% (*Q.exilispinosa*), which was greater than that between *Q.exilispinosa* and *Q.acanthophora* (6.20–6.75%); between *Q.acanthophora* and *Q.spinosa* (7.82–8.93%); between *Q.exilispinosa* and *Q.spinosa* (7.65–8.02%) (Table 3). The ML and BI analyses produced topologies with –ln L = 4069.066 and 4137.061, respectively, with a gamma shape parameter (G: 0.195 in ML and 0.185 in BI). Phylogenetic analyses employing ML and BI methods were nearly identical, with most well-supported nodes on the ML tree also well-supported on the BI tree, and only the BI tree is presented in Fig. 2.

**Table 3.** Uncorrected *p*-distance matrix showing percentage pairwise genetic divergences (%) for the Cytb rRNA gene between members of the genus *Quasipaa*.

		1	2	3	4	5	6	7	8	9	10	11	12	13	14
1	*Quasipaaohlerae* sp. nov.	**0.00–0.83**													
2	*Q.delacouri*	13.81–14.21	**0.00**												
3	*Quasipaabinhi* sp. nov.	15.87–16.45	10.15	**0.00**											
4	Q.cf.boulengeri	21.22–21.26	20.20	21.25	**0.00**										
5	*Q.boulengeri*	19.58–21.16	18.82–19.36	20.58–21.88	11.72–12.96	**0.00–4.30**									
6	*Quasipaa* sp.	22.20–22.91	19.11–19.34	21.40	15.06–16.08	15.83–18.56	**0.00–1.50**								
7	*Q.verrucospinosa*	21.50–22.26	18.00–18.73	18.28–19.99	15.88–16.17	15.41–17.97	12.75–13.75	**0.00–0.50**							
8	*Q.shini*	19.71–20.18	20.66	20.93	16.71	18.59–19.64	18.42–18.91	18.33–18.79	**0.00**						
9	*Q.yei*	19.88–20.35	18.61	19.97	19.10	20.82–22.38	18.47–19.13	18.11–18.40	19.92	**0.00**					
10	*Q.acanthophora*	20.15–21.77	19.31–20.37	19.79–20.44	17.44–18.32	18.11–19.69	17.51–18.57	17.46–18.54	16.73–16.97	17.28–17.52	**0.00–1.33**				
11	*Q.spinosa*	21.68–22.39	20.26–20.48	21.29–21.60	18.97–19.18	20.51–21.71	22.11–22.74	21.62–21.92	18.35–18.56	19.60–19.82	7.82–8.93	**0.00–0.16**			
12	*Q.exilispinosa*	19.23–19.69	21.20–21.42	21.35–21.66	18.18–18.39	19.55–21.40	19.31–19.76	19.57–19.98	18.35–18.56	16.62–16.83	6.20–6.75	7.65–8.02	**0.00–0.16**		
13	*Q.jiulongensis*	17.61–18.28	18.51–18.72	20.52	19.12–19.33	17.67–18.40	18.90–19.56	18.32–19.00	17.63–17.84	18.64–18.86	10.94–11.54	12.26–12.64	12.70–13.09	**0.00–0.50**	
14	*Q.taoi*	18.38–19.52	16.92–17.58	19.93–19.96	18.56–19.27	17.80–19.70	17.53–17.55	19.68–20.59	17.77–18.23	17.35–17.98	14.83–15.47	16.75–17.36	16.97–17.39	17.06–17.72	**0.00–1.00**

Both analyses on the two 16S and Cytb rRNA segments are relatively similar, especially the separation in clades corresponding to separate species (Figs 1, 2) with significant bootstrap values. *Quasipaaohlerae* sp. nov.is genetically sister to *Quasipaabinhi* sp. nov. and *Q.delacouri* with a strong nodal support from both analyses (1/94.7 on 16S; 1/99.4 on Cytb). *Quasipaaverrucospinosa* sensu stricto genetically sister to *Quasipaa* sp. (mentioned in Yan et al. 2021). *Quasipaaboulengeri* is genetically sister to both species, *Quasipaa* sp. and *Q.verrucospinosa*. *Quasipaajiulongensis* are genetically sister species to *Q.exilispinosa*, *Q.spinosa* and *Q.acanthophora*. Both analysis on the two 16S and Cytb gene segments differ only by site of *Q.yei* and *Q.shini* in phylogenetic trees.

Our phylogenetic results confirmed phylogenetic results of the genus *Quasipaa* from previous studies (Che et al. 2009, 2010; Yan et al. 2021). In addition, the specimens collected from northern Vietnam (Tuyen Quang, Ha Giang, and Bac Kan provinces) and other specimens collected from the type localities (Sa Pa in Lao Cai Province and Tam Dao in Vinh Phuc Province) are embedded in the same clade with *Q.verrucospinosa* sensu stricto. The specimens previously assigned to *Q.verrucospinosa* from Thailand by Suwannapoom et al. (2021) and the specimens from Laos (Phongsaly Province), China (Yunnan Province) and central Vietnam (Nghe An Province) are clustered in the same clade with the new species (Figs 1, 2).

### ﻿Morphological analysis

**Figure 3.**
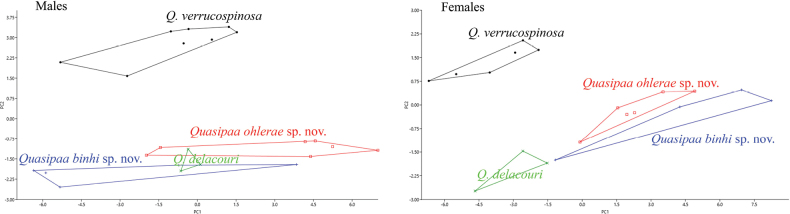
Plots of the first principal component (PC1) versus the second (PC2) for the males and the females of *Quasipaaohlerae* sp. nov. (red square), *Quasipaabinhi* sp. nov. (blue plus symbol), *Q.delacouri* (green x), and *Q.verrucospinosa* (black dot).

The first two principal component axes could separate the females of two new species from *Quasipaaverrucospinosa* and *Q.delacouri* by 22 characteristics SVL, HW, HL, MN, MFE, MBE, RL, ED, UEW, IND, IOD, DAE, DPE, NS, EN, UAL, FAL, FeL, TbL, TbW, FoL, and IMT (Table 4, Fig. 3). In females, the PCA extracted three principal component axes with eigenvalues greater than 0.70, the first two component axes accounted for 89.71% of the variation (Table 4). The PC1 of species with positive scores were associated with species having greater measurements of 21 characters (SVL, HW, HL, MN, MFE, MBE, RL, ED, UEW, IND, IOD, DAE, DPE, NS, EN, FAL, FeL, TbL, TbW, FoL, and IMT) and negative score were associated with species having smaller measurements of 1 character UAL. Species with a higher and positive score on PC2 reflected having shorter measurements of HW, RL, IOD, DPE, NS, EN, UAL and TbW; while a negative score with species having smaller SVL, HL, MN, MFE, MBE, ED, UEW, IND, FAL, FeL, TbL, FoL, and IMT. (Table 4). The first two principal component axes could separate the males of two new forms from *Quasipaaverrucospinosa* by 22 characters SVL, HW, HL, MN, MFE, MBE, RL, ED, UEW, IND, IOD, DAE, DPE, NS, EN, UAL, FAL, FeL, TbL, TbW, FoL, and IMT (Table 4, Fig. 3). Especially, the UAL character has a strong influence on the morphological separation between male specimens of the species. In males, the PCA extracted three principal component axes with eigenvalues greater than 1.01, the first two component axes accounted for 83.47% of the variation (Table 4). Species with the PC1 with positive scores were associated with species having greater measurements of 22 characters. Species with a higher and positive score on PC2 reflected having shorter measurements of SVL, HW, HL, RL, IOD, DPE, EN, UAL, FAL, FeL, TbL, TbW, FoL, and IMT; while a negative score with species having smaller MN, MFE, MBE, ED, UEW, IND, DAE, and NS (Fig. 3).

**Table 4.** Variable loadings for principal components with eigenvalue greater than 0.70, from morphometric characters corrected by SVL. All measurements were given in millimeter (mm).

	Male			Female		
	PC 1	PC 2	PC 3	PC 1	PC 2	PC 3
Eigenvalue	13.66	4.71	1.01	18.02	1.72	0.70
variance	62.08	21.39	4.61	81.91	7.80	3.20
SVL	0.24	0.14	0.03	0.23	-0.04	0.19
HL	0.24	0.13	-0.17	0.22	-0.02	-0.06
HW	0.23	0.21	-0.07	0.23	0.09	0.09
MN	0.20	-0.28	-0.18	0.23	-0.06	-0.23
MFE	0.19	-0.31	-0.02	0.23	-0.03	-0.20
MBE	0.16	-0.35	0.01	0.22	-0.07	-0.19
RL	0.24	0.09	0.11	0.22	0.20	-0.12
ED	0.21	-0.23	0.08	0.21	-0.06	0.12
UEW	0.19	-0.26	-0.12	0.22	-0.05	-0.26
IND	0.21	-0.10	0.30	0.22	-0.06	0.00
IOD	0.14	0.33	0.10	0.14	0.55	0.27
DAE	0.21	-0.24	-0.01	0.23	-0.07	-0.23
DPE	0.25	0.07	-0.12	0.23	0.09	0.02
NS	0.17	-0.01	0.61	0.20	0.31	-0.17
EN	0.19	0.03	-0.60	0.20	0.23	-0.38
UAL	0.00	0.46	-0.08	-0.13	0.62	0.03
FAL	0.23	0.20	0.00	0.22	-0.03	0.23
FeL	0.24	0.13	-0.10	0.22	-0.07	0.07
TbL	0.26	0.02	-0.01	0.23	-0.15	0.13
TbW	0.22	0.20	0.18	0.21	0.12	0.31
FoL	0.26	0.04	0.10	0.22	-0.17	0.08
IMT	0.24	0.01	0.00	0.20	-0.12	0.49

### ﻿Taxonomic accounts

In the following, based on the distinct molecular divergence in concert with diagnostic morphological differences compared to congeners, we describe *Quasipaaohlerae* sp. nov from the provinces of Son La, Thanh Hoa, Nghe An and *Quasipaabinhi* sp. nov. from Quang Binh, Thua Thien-Hue provinces of Vietnam, as two new species. Simultaneously, we redescribe *Q.verrucospinosa* sensu stricto based on specimens collected from Vinh Phuc (Tam Dao) and Lao Cai (Sa Pa) provinces of Vietnam.

#### ﻿Class Amphibia Linnaeus, 1758

**Order Anura Hogg, 1839**

**Family Dicroglossidae Anderson, 1871**

**Genus *Quasipaa* Dubois, 1992**

##### ﻿ Quasipaa verrucospinosa

Taxon classificationAnimaliaAnuraDicroglossidae

(Bourret, 1937)

C9F126A2-CD82-57E3-88FF-4292FE359B20

**Figure 4.**
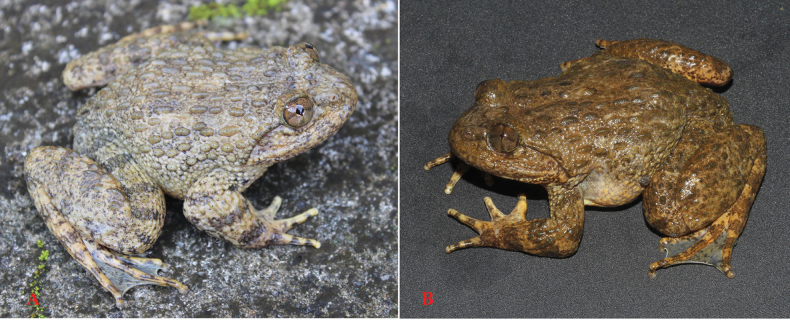
*Quasipaaverrucospinosa* in life **A** male (IEBR A.5153) **B** female (IEBR A.5156).

**Figure 5.**
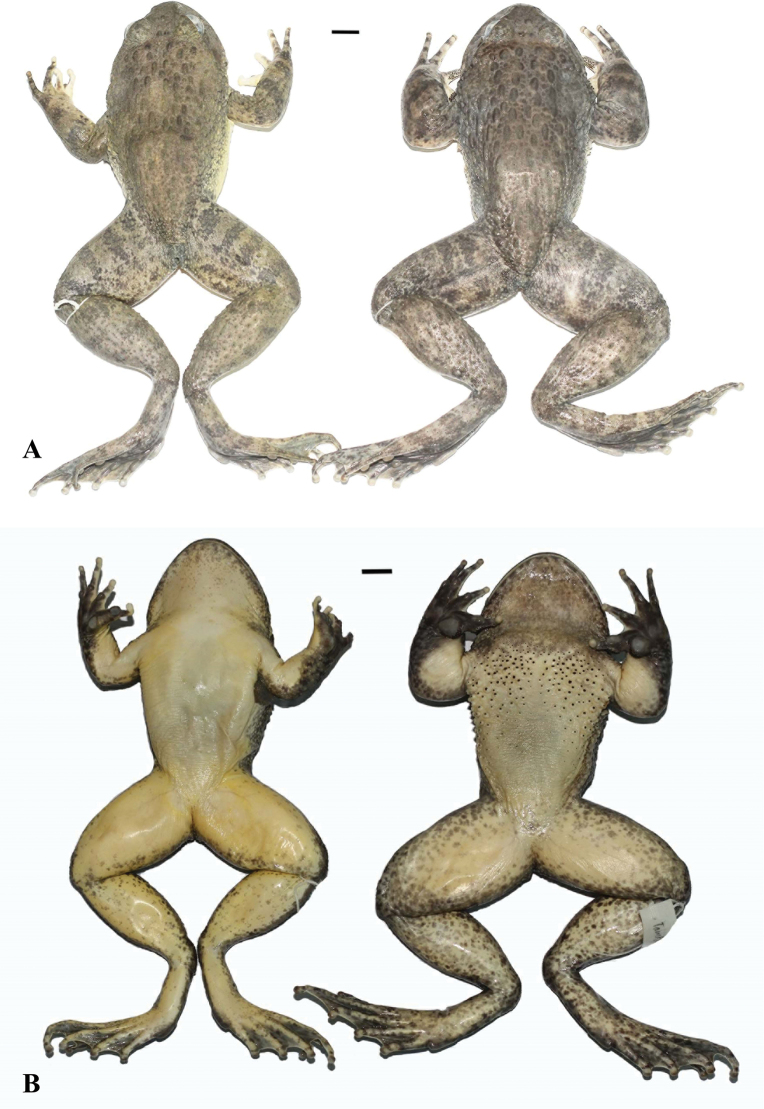
*Quasipaaverrucospinosa* (IEBR A.5156, female and IEBR A.5153, male) in preservative **A** dorsolateral view **B** ventral view. Scale bars: 10 mm

**Figure 6.**
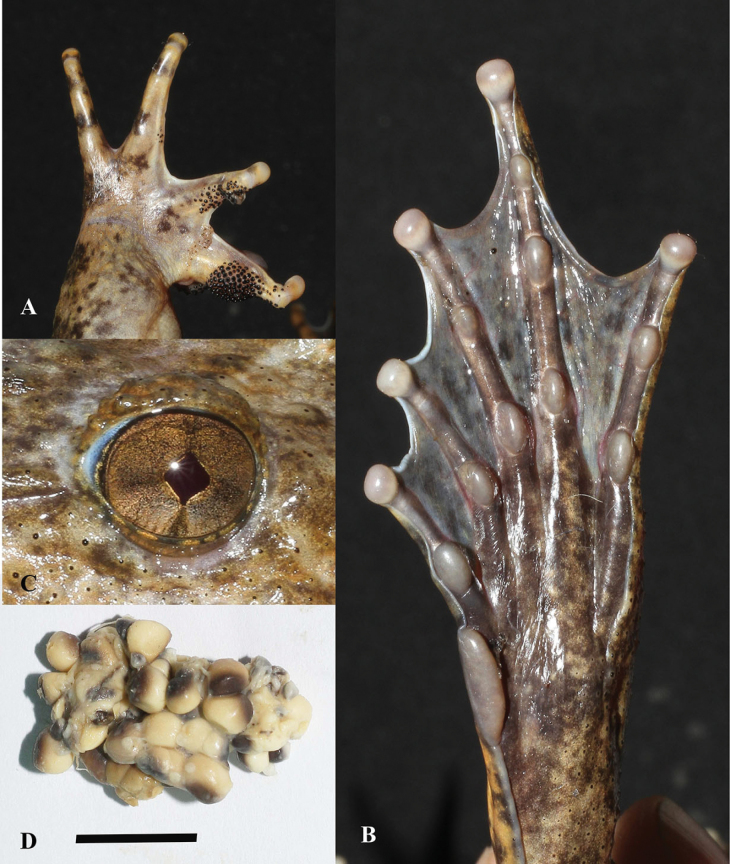
*Quasipaaverrucospinosa***A** upper left hand of male (IEBR A.5153) **B** lower right foot of male (IEBR A.5153) **C** iris of male (IEBR A.5153) **D** eggs of female (IEBR A.5156). Scale bar: 10 mm

Figs 4 , 5 , 6 , Table 5

**Figure 7.**
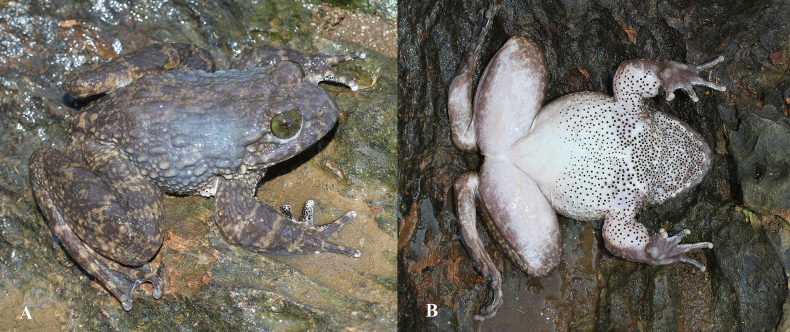
*Quasipaaohlerae* sp. nov., holotype (IEBR A.5159, male) in life **A** dorsolateral view **B** ventral view.

Rana spinosa verrucospinosa Bourret, 1937: 8., fig. 7.
Rana verrucospinosa : Bourret 1942: 295–296, fig. 83.
Paa verrucospinosa : Inger et al. 1999: 22–23.
Quasipaa verrucospinosa : Ohler and Dubois 2006: 781.
Quasipaa cf. verrucospinosa 1: Suwannapoom et al. 2021: 1–12.

###### Material examined.

(*n* = 14) • IEBR A.5153–5155, three adult males and IEBR A.5021, 5156, two adult females, collected by C. T. Pham, on 18 July 2022, in Tam Dao National Park (21°27.507'N, 105°38.874'E, at an elevation of 985 m a.s.l.), Vinh Phuc Province, Vietnam; • IEBR A.5023, 5024, two adult males and IEBR A.5022, adult female, collected by C. V. Hoang, on 23 November 2015, in Bac Me Nature Reserve (22°49.976'N, 105°07.648'E, at an elevation of 780 m a.s.l.), Ha Giang Province, Vietnam; • IEBR A.5028, adult male and IEBR A.5026, 5027, two adult females, collected by C. T. Pham and T. Q. Phan, on 25 August 2017, in Sinh Long Commune (22°34.288'N, 105°20.119'E, at an elevation of 801 m a.s.l.), Na Hang District, Tuyen Quang Province; • IEBR A.5025, adult female, collected by C. T. Pham, C. V. Hoang, and T. Q. Phan, on 27 October 2018, in Cham Chu Nature Reserve (22°12.494'N, 105°04.423'E; at an elevation of 981 m a.s.l.), Ham Yen District, Tuyen Quang Province; • IEBR A.5157, adult male and IEBR A.5158, adult female, collected by C. T. Pham, on 12 October 2022, in Hoang Lien National Park (22°09.557'N, 104°04.194'E; at an elevation 2,078 m a.s.l.), Sa Pa District, Lao Cai Province, Vietnam.

###### Revised diagnosis.

(1) A large frog (SVL up to 106 mm in males and 95 mm in females); (2) head broader than long (HL/HW 0.88 in males, 0.89 in females); (3) vomerine teeth present; (4) external vocal sacs absent; (5) tympanum visible, round; (6) dorsum with thick ridges and small round tubercles; (7) flanks covered by oval and round tubercles; (8) supratympanic fold present; (9) dorsolateral fold absent; (10) males with dense spines on chest, 2/3 anterior part of belly and fingers I, II, III (sometimes present on fingers I and II only); (11) male without spines on finger IV and ventral surface of forelimbs; (12) finger I with nuptial pad in males; (13) yellowish cream eggs with melanic poles in females; (14) toes fully webbed to distal end of terminal phalanx; and (15) in life, dorsum yellowish grey or pale brownish grey, chest and belly pale yellowish white, iris pale copper.

###### Description.

A large frog (SVL up to 106 mm in males and 95 mm in females); habitus robust with enlarged head (HL/SVL 0.38 ± 0.01, HW/SVL 0.43 ± 0.01, in males and HL/SVL 0.38 ± 0.01, HW/SVL 0.43 ± 0.01, in females); head broader than long (HL 38.0 ± 2.8 mm, HW 42.9 ± 3.0 mm, in males and HL 32.8 ± 2.0 mm, HW 37.0 ± 2.2 mm, in females); snout round anteriorly in dorsal view, projecting beyond lower jaw; nostril lateral, closer to eye than to the tip of snout; canthus rostralis indistinct; loreal region oblique and slightly concave; rostral length greater than eye diameter; internarial distance wider than interorbital distance and upper eyelid width; tympanum slightly visible (TD 5.4 ± 0.5 mm, in males and 5.0 ± 0.5 mm, in females) smaller than the distance from tympanum to eye (TYE 6.3 ± 0.6 mm, in males and 5.6 ± 0.7 mm, in females), ~ 50% eye diameter; vomerine teeth in two oblique ridges; tongue cordiform, notched posteriorly; external vocal sac absent.

***Forelimbs***: arms short; upper arm length (UAL 20.1 ± 2.8 mm, in males and UAL 15.5 ± 1.4 mm, in females), forearm length (FAL 48.5 ± 3.5 mm, in males and FAL 38.4 ± 2.6 mm, in females); relative finger lengths: II<I<IV<III; fingers free of webbing; sides of fingers I, II, and III with narrow dermal ridge; tips of fingers swollen, not expanded; subarticular tubercles prominent, round, formula 1, 1, 2, 2; inner metatarsal tubercle round; outer metatarsal tubercle elongate; finger I with nuptial pad in males.

***Hindlimbs***: tibia length longer than thigh length (FeL 52.0 ± 3.1 mm, TbL 53.1 ± 3.0 mm, in males and FeL 44.8 ± 3.3 mm, TbL 45.7 ± 3.1 mm, in females), ~ 3× longer than wide (TbW 18.9 ± 1.8 mm, in males and TbW 15.0 ± 1.7 mm, in females); tips of toes swollen, round; relative length of toes: I<II<V<III<IV; toes fully webbed to distal end of terminal phalanx; dermal ridge present on outer sides of toes I and V; subarticular tubercles prominent, oval, formula 1, 1, 2, 3, 2; inner metatarsal tubercle elongate; outer metatarsal tubercle absent; tibio-tarsal articulation reaching to nostril.

***Skin texture in life***: dorsal surface of head with oval and round tubercles, dorsum with thick ridges intermixed with small round tubercles; flanks covered by oval and round tubercles; supratympanic fold distinct, extending from eye to angle of jaw; dorsolateral fold absent; dorsal surface of forelimb and hindlimb with small tubercles; belly and ventral surface of thigh smooth.

***Nuptial spines***: body of males with spines; dense spines on lower flanks, chest, 2/3 anterior part of belly and fingers I, II, III; spines present on dorsum, upper flanks, upper lip, dorsal surface of fore- and hindlimbs, lower lip, and throat small and scattered; spines absent on finger IV and ventral surface of forelimbs.

***Coloration in life***: iris pale copper; dorsum and upper part of flanks yellowish grey or pale brownish grey; lower part of flanks whitish yellow with white tubercles and black spines on top; dorsal surface of limbs yellowish with brown crossbars; ventral surface of limbs yellowish white; throat white with brown markings; chest and belly pale yellowish white; toe webbing pale brown.

###### Sexual dimorphism.

Measurements and morphological characters of the *Quasipaaverrucospinosa* are provided in Table 5. males are slightly larger than females (SVL 100.7 ± 6.6 mm, *n* = 7 males vs 86.8 ± 7.4 mm, *n* = 7 females). The male specimens have a nuptial pad on finger I and dense spines on lower part of flanks, chest, 2/3 anterior part of belly, and fingers I, II, III. The females contained yellowish cream eggs with melanic poles.

**Table 5.** Measurements (in mm) and proportions of *Quasipaaverrucospinosa* (M = Male, F = Female, SD = standard deviation; for other abbreviations see Material and methods).

	*Quasipaaverrucospinosa*				*Quasipaadelacouri*			
Voucher	Min–Max (*n* = 7)	Mean ± SD (*n* = 7)	Min–Max (*n* = 7)	Mean ± SD (*n* = 7)	Min–Max (*n* = 3)	Mean ± SD (*n* = 3)	Min–Max (*n* = 3)	Mean ± SD (*n* = 3)
Sex	M	M	F	F	M	M	F	F
SVL	84.5–105.5	100.7 ± 6.6	75.2–94.7	86.8 ± 7.4	95.9–104.9	99.8 ± 4.6	90.5–98.4	94.1 ± 4.0
HL	31.1–39.9	38 ± 2.8	29.6–34.9	32.8 ± 20	33.5–35.6	34.3 ± 1.1	33.4–36.5	35.17 ± 1.6
HW	35.7–44.7	42.9 ± 3	33.3–39.6	37 ± 2.2	33.5–37.6	36.7 ± 1.0	34.6–40.0	37,1 ± 2.7
MN	21.9–27.3	26 ± 2	21.6–25.4	23.7 ± 1.3	27.8–28.9	28.4 ± 0.6	23.9–29.5	27.2 ± 2.9
MFE	17.7–21.1	20.3 ± 1.1	16.6–20.1	18.6 ± 1.1	22.7–24.7	23.6 ± 1.0	17.8–25.7	22.4 ± 4.1
MBE	10.2–13.4	11.7 ± 1.1	8.6–11.8	10.5 ± 1	15.6–16.8	16.0 ± 0.7	14.5–17.4	15.7 ± 1.5
RL	13.2–15.4	15.1 ± 0.7	11.9–14.2	13.1 ± 1	13.2–14.7	14.1 ± 0.8	11.5–15.2	13.7 ± 1.9
ED	8.6–10.8	10.3 ± 0.7	9–11.5	10 ± 1	11.3–12.9	11.9 ± 0.8	11.1–12.9	12.3 ± 1.0
UEW	6.5–8.4	7.7 ± 0.6	6.0–7.5	7.0 ± 0.6	7.9–9.6	8.8 ± 0.9	7.1–8.8	7.8 ± 0.9
IND	7.3–9.7	8.7 ± 0.8	6.0–8.4	7.4 ± 0.9	10.2–11.2	10.6 ± 0.5	8.9–10.9	10.0 ± 1.0
IOD	6.9–8.9	8.2 ± 0.6	6.2–7.9	7.3 ± 0.6	6.2–7.3	6.8 ± 0.6	6.3–7.7	7.1 ± 0.7
DAE	12.1–14.6	13.5 ± 1	11.3–13.8	12.4 ± 0.9	15.5–6.3	15.9 ± 0.4	13.9–16.8	15.5 ± 1.5
DPE	22.8–28.2	26.3 ± 1.6	21.3–25.9	23.9 ± 1.6	24.5–24.9	24.7 ± 0.8	23.2–25.6	24.4 ± 1.2
NS	6.0–9.0	7.5 ± 0.9	6.1–8.1	7.0 ± 0.8	7.7–8.1	7.9 ± 0.2	6.5–8.8	7.7 ± 1.2
EN	5.7–7.4	6.8 ± 0.6	5.5–6.4	6.0 ± 0.3	5.8–6.6	6.3 ± 0.4	5.8–7.2	6.4 ± 0.7
TD	4.8–6.3	5.4 ± 0.5	4.6–5.9	5.0 ± 0.5	3.0–3.2	3.1 ± 0.1	2.8–3.1	2.9 ± 0.2
TYE	5.1–6.8	6.3 ± 0.6	4.6–6.6	5.6 ± 0.7	6.4–6.9	6.7 ± 0.3	5.5–5.8	5.6 ± 0.2
UAL	13.0–21.0	20.1 ± 2.8	13.4–16.7	15.5 ± 1.4	14.3–15.4	14.8 ± 0.5	14.5–16.2	15.4 ± 0.8
FAL	40.2–50.2	48.5 ± 3.5	34.2–41.9	38.4 ± 2.6	41.1–43.3	42.4 ± 1.2	40.4–44.5	41.8 ± 2.3
FeL	47.2–55.3	52.0 ± 3.1	38.4–49.3	44.8 ± 3.8	46.7–48.9	47.7 ± 1.1	46.9–49.8	49.1 ± 1.5
TbL	47.0–55.9	53.1 ± 3	40.4–48.9	45.7 ± 3.1	50.2–53.8	51.6 ± 1.9	50.0–54.7	51.5 ± 2.4
TbW	16.3–21.9	18.9 ± 1.8	12.8–17.3	15.0 ± 1.7	17.3–17.7	17.5 ± 0.2	14.9–17.2	15.8 ± 1.3
FoL	62.9–73.6	70.7 ± 3.5	54.7–65.5	60.8 ± 4.2	67.5–73.1	70.4 ± 2.8	68.5–71.3	69.7 ± 1.4
IMT	7.5–9.5	8.9 ± 0.7	5.4–8.1	6.9 ± 1	8.3–8.5	8.4 ± 0.1	7.8–9.0	8.5 ± 0.6
HL/SVL	0.37–0.39	0.38 ± 0.01	0.36–0.39	0.38 ± 0.01	0.32–0.36	0.34 ± 0.02	0.37–0.38	0.37 ± 0.01
HW/SVL	0.41–0.44	0.43 ± 0.01	0.41–0.44	0.43 ± 0.01	0.35–0.38	0.37 ± 0.02	0.38–0.41	0.39 ± 0.02
RL/SVL	0.14–0.16	0.15 ± 0.01	0.14–0.16	0.15 ± 0.01	0.13–0.15	0.14 ± 0.01	0.13–0.15	0.14 ± 0.01
HL/HW	0.85–0.91	0.88 ± 0.02	0.86–0.91	0.89 ± 0.02	0.92–0.95	0.94 ± 0,02	0.91–0.97	0.95 ± 0.03
ED/RL	0.65–0.72	0.69 ± 0.03	0.65–0.85	0.77 ± 0.08	0.80–0.89	0.85 ± 0.05	0.85–0.97	0.90 ± 0.06
TYE/TD	0.94–1.34	1.16 ± 0.14	0.95–1.37	1.14 ± 0.16	2.00–2.16	2.15 ± 0.15	1.84–2.00	1.93 ± 0.08
TD/ED	0.46–0.60	0.53 ± 0.05	0.41–0.60	0.50 ± 0.06	0.23–0.27	0.26 ± 0.02	0.22–0.25	0.24 ± 0.01
TbL/SVL	0.51–0.56	0.53 ± 0.01	0.51–0.56	0.53 ± 0.02	0.48–0.55	0.55 ± 0,04	0.55–0.57	0.56 ± 0.01
TbL/TbW	2.56–3.00	2.81 ± 0.15	2.78–3.34	3.06 ± 0.18	2.85–3.04	2.94 ± 0.09	3.18–3.48	3.34 ± 0.15

###### Distribution.

The species was recorded in Lao Cai (Hoang Lien National Park), Vinh Phuc (Tam Dao National Park), Ha Giang (Bac Me Nature Reserve), and Tuyen Quang (Na Hang Nature Reserve and Cham Chu Nature Reserve) provinces, northern Vietnam.

###### Remarks.

The specimens agreed well with the descriptions of Bourret (1942) and Inger et al. (1999) in size, skin texture, and coloration; males with dense spines on 2/3 anterior part of belly. In addition, dorsum yellowish grey and belly pale yellowish white (more yellow in females); males with spines on fingers I, II, and III (some times present on fingers I and II only); yellowish cream eggs with melanic poles in females; iris pale copper.

##### ﻿ Quasipaa ohlerae sp. nov.

Taxon classificationAnimaliaAnuraDicroglossidae

0A83E8F4-0FF5-5FB3-A0D4-CB2DDB9125C1

https://zoobank.org/771C9CEA-691F-4B12-8BAC-78974B998F49

**Figure 8.**
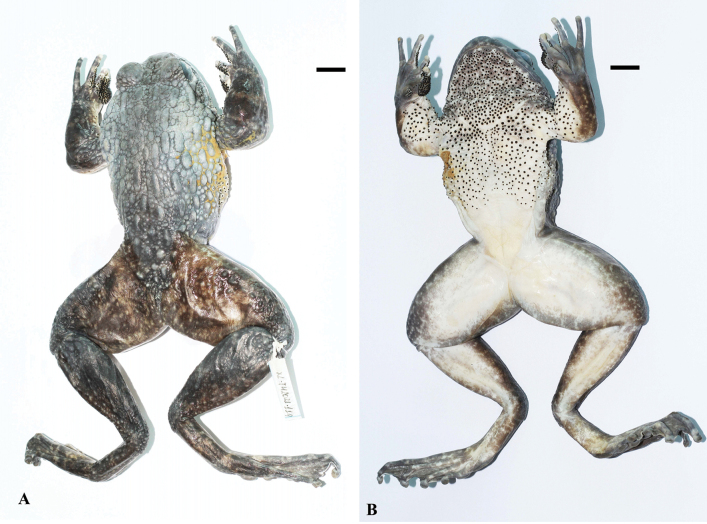
*Quasipaaohlerae* sp. nov., holotype (IEBR A.5159, male) in preservative **A** dorsolateral view **B** ventral view. Scale bars: 10 mm

**Figure 9.**
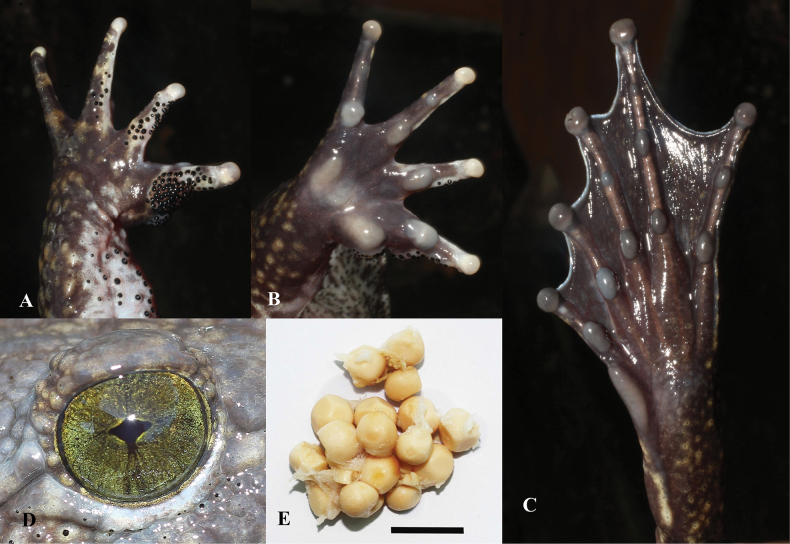
*Quasipaaohlerae* sp. nov. **A** upper left hand of holotype (IEBR A.5159, male) **B** lower right hand of holotype (IEBR A.5159, male) **C** lower right foot of holotype (IEBR A.5159, male) **D** iris of holotype (IEBR A.5159, male) **E** eggs of paratype (IEBR A.5160, female). Scale bar: 10 mm

**Figure 10.**
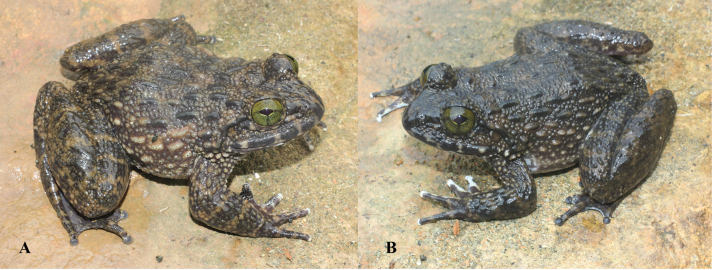
*Quasipaaohlerae* sp. nov., paratypes in life **A** dorsolateral view (IEBR A.5161, male) **B** dorsolateral view (IEBR A.5160, female).

**Figure 11.**
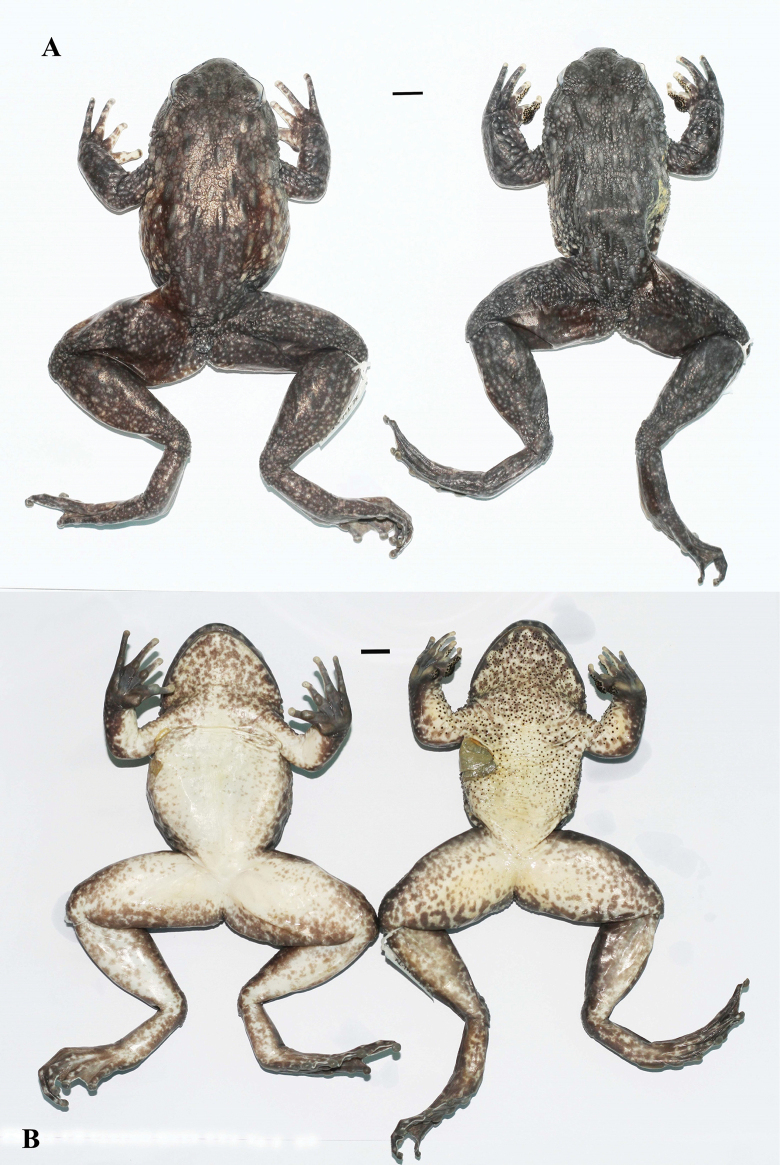
*Quasipaaohlerae* sp. nov., paratypes (IEBR A.5160, female and IEBR A.5161, male) in preservative **A** dorsolateral view **B** ventral view. Scale bars: 10 mm

Figs 7 , 8 , 9 , 10 , 11 , Table 6

Paa verrucospinosa : Hu et al. 2005: 340–341.
Quasipaa verrucospinosa : Yan et al. 2021: 1–7. Suwannapoom et al. 2021: 1–12, fig. 3.
Quasipaa cf. verrucospinosa : Dau et al. 2024: 9–11, fig. 5.

###### Type material.

***Holotype*.** • IEBR A.5159, adult male, collected by T. Q. Phan and H. Q. Nguyen, on 17 November 2021, in Xuan Lien Nature Reserve (19°59.076'N, 104°59.095'E, at an elevation of 806 m a.s.l.), Thuong Xuan District, Thanh Hoa Province, Vietnam. ***Paratypes*.** (*n* = 12) • IEBR A.5160, adult female, collected by T. Q. Phan and C. V. Hoang, on 20 October 2021, in Xuan Lien Nature Reserve (19°52.041'N, 105°12.569'E, at an elevation of 297 m a.s.l.), Thuong Xuan District, Thanh Hoa Province, Vietnam; IEBR A.5161–5163, three adult males and IEBR A.5164–5166, three adult females, collected by C. T. Pham and C. V. Hoang, on 25 August 2012, in Xuan Lien Nature Reserve (19°51.446'N, 105°12.153'E, at an elevation of 423 m a.s.l.), Thuong Xuan District, Thanh Hoa Province, Vietnam; ZVNU 11, adult male, collected by A. V. Pham, on 22 December 2012, in Copia Nature Reserve (21°20.216'N, 103°34.822'E, at an elevation of 950 m a.s.l.), Thuan Chau District, Son La Province, Vietnam; ZVNU 12, adult male, collected by T. Q. Nguyen, A. V. Pham, and H. N. Ngo, on 17 September 2014, in Copia Nature Reserve (21°20.216'N, 103°34.822'E, at an elevation of 950 m a.s.l.), Thuan Chau District, Son La Province, Vietnam; ZVNU 14, adult male and ZVNU 13, adult female, collected by A. V. Pham and N. B. Sung, on 16 September 2016, in Copia Nature Reserve (21°20.216'N, 103°34.822'E, at an elevation of 950 m a.s.l.), Thuan Chau District, Son La Province, Vietnam; IEBR A.5167, adult female, collected by A. V. Ong, on 26 October 2021, in Pu Hoat Nature Reserve (19°44.245'N, 104°57.474'E, at an elevation of 655 m a.s.l.), Que Phong District, Nghe An Province, Vietnam.

###### Diagnosis.

Both morphological characteristics (body very stout, skin rough with dermal ridges and tubercles, forelimbs of males strongly enlarged, with inner side of arms, fingers or chest and belly with black spines) (Fei et al. 2009) and molecular data revealed the new species to be nested within *Quasipaa*. *Quasipaaohlerae* sp. nov. is distinguishable from its congeners by a combination of the following morphological characteristics: (1) SVL 86.7–107.8 mm in males and 92.7–107.0 mm in females; (2) head broader than long (HL/HW 0.89 in males, 0.88 in females); (3) vomerine teeth present; (4) external vocal sacs absent; (5) tympanum visible, round; (6) dorsum with thick ridges and small round tubercles; (7) flanks covered by oval and round tubercles; (8) supratympanic fold present; (9) dorsolateral fold absent; (10) ventral surface of arms and all fingers with spines in males; (11) fingers I and II with nuptial pad in males; (12) each chest tubercle with one black spine in males; (13) females with yellowish cream eggs; (14) toes fully webbed to distal end of terminal phalanx; and (15) in life, dorsum dark brown, chest and belly immaculate white, iris dark green.

###### Description of holotype.

A large frog (SVL 103.1 mm); habitus robust with enlarged head (HL/SVL 0.38, HW/SVL 0.43); head broader than long (HL 39.4 mm, HW 44.6 mm); snout round anteriorly in dorsal view, projecting beyond lower jaw; nostril lateral, closer to eye than to the tip of snout (NS 9.0 mm, EN 7.9 mm); canthus rostralis indistinct; loreal region oblique and slightly concave; rostral length greater than eye diameter (RL 16.0 mm, ED 13.0 mm); internarial distance wider than interorbital distance and upper eyelid width (IND 10.2 mm, IOD 7.4 mm, UEW 9.3 mm); tympanum visible (TD 4.0 mm) smaller than the distance from tympanum to eye (TYE 7.0 mm), ~ 30% eye diameter; vomerine teeth in two oblique ridges; tongue cordiform, notched posteriorly; external vocal sac absent.

***Forelimbs***: arms short; upper arm length (UAL) 23.1 mm, forearm length (FAL) 51.0 mm; relative finger lengths: II<I<IV<III; fingers free of webbing; narrow dermal ridge on sides of fingers present on fingers II, III; tips of fingers swollen, not expanded; subarticular tubercles prominent, round, formula 1, 1, 2, 2; inner metatarsal tubercle oval; outer metatarsal tubercle elongate; fingers I and II with nuptial pad.

***Hindlimbs***: tibia length longer than thigh length (FeL 52.8 mm, TbL 60.3 mm), ~ 3.2× longer than wide (TbW 19.1 mm); tips of toes swollen, slightly round; relative length of toes: I<II<V<III<IV; toes fully webbed to distal end of terminal phalanx; dermal ridge present on outer sides of toes I and V; subarticular tubercles prominent, elongate, formula 1, 1, 2, 3, 2; inner metatarsal tubercle elongate; outer metatarsal tubercle absent; tibio-tarsal articulation reaching to nostril.

***Skin texture in life***: dorsal surface of head with oval and round tubercles, dorsum with thick ridges intermixed with small round tubercles; flanks covered by oval and round tubercles; supratympanic fold distinct, extending from eye to angle of jaw; dorsolateral fold absent; dorsal surface of forelimbs and hindlimbs with small tubercles; belly and ventral surface of thighs smooth.

***Nuptial spines***: body of males with spines except for ~ 1/3 posterior part of belly and ventral surface of hindlimbs; dense spines on lower flanks, ventral surface of forelimbs, lower lip, throat, chest, 2/3 anterior part of belly and fingers I, II, III; dorsum, upper flanks, upper lip, dorsal surface of fore- and hindlimbs, and finger IV with small spines, scattered; each chest tubercle with one black spine.

***Coloration in life***: iris dark green; dorsum and upper part of flanks dark brown; lower part of flanks whitish brown with white tubercles and black spines on top; dorsal surface of limbs yellowish brown with dark crossbars; throat white with brown markings; ventral surface of limbs, chest, and belly immaculate white; toe webbing dark brown.

***Coloration in preservative***: coloration in preservative is the same in life but somewhat faded.

###### Sexual dimorphism.

Measurements and morphological characteristics of the type series are provided in Table 6. The male specimens have a nuptial pad on fingers I and II and black spines on lower flanks, ventral surface of forelimbs, lower lip, throat, chest, 2/3 anterior part of belly, and all fingers. The females contained yellowish cream eggs, wholly unpigmented.

**Table 6.** Measurements (in mm) and proportions of the type series of *Quasipaaohlerae* sp. nov. (H = holotype, P = paratype, M = Male, F = Female, SD = standard deviation; for other abbreviations see Materiasl and methods).

Voucher	IEBR A.5159	IEBR A.5162	IEBR A.5161	IEBR A.5163	ZVNU 11	ZVNU 14	ZVNU 12	Min–Max (*n* = 7)	Mean ± SD (*n* = 7)	IEBR A.5166	IEBR A.5165	IEBR A.5164	IEBR A. 5167	ZVNU 13	IEBR A.5160	Min–Max (*n* = 6)	Mean ± SD (*n* = 6)
Sex	M	M	M	M	M	M	M			F	F	F	F	F	F		
Type status	H	P	P	P	P	P	P			P	P	P	P	P	P		
SVL	103.1	103.8	86.7	87.4	107.8	104.6	100.5	86.7–107.8	99.1 ± 8.5	99.7	97.8	92.7	97.1	107.0	103.2	92.7–107.0	99.6 ± 5.0
HL	38.5	38.2	34.7	34.8	40.1	39.8	39.0	34.7–40.1	37.9 ± 2.2	37.1	36.7	33.2	37.7	40.0	39.2	33.2–40.0	37.3 ± 2.4
HW	44.6	42.9	38.1	37.8	45.5	45.0	43.6	37.8–45.5	42.5 ± 3.2	42.7	42.1	37.8	41.8	45.0	43.6	37.8–45.0	42.2 ± 2.4
MN	32.9	33.9	29.5	29.3	34.4	33.0	33.0	29.3–34.4	32.3 ± 2.1	31.7	31.3	28.7	32.8	33.2	34.0	28.7–34.0	31.9 ± 1.9
MFE	28.0	28.7	23.8	24.7	29.2	27.2	27.6	23.8–29.2	27.0 ± 2.0	25.6	26.2	24.1	23.4	27.3	28.8	23.4–28.8	25.9 ± 2.0
MBE	17.0	15.8	15.2	15.4	18.5	18.0	17.2	15.2–18.5	16.7 ± 1.3	15.7	15.8	16.4	16.6	17.0	18.5	15.7–18.5	16.7 ± 1.0
RL	16.0	14.8	13.4	13.7	17.0	16.7	16.2	13.4–17.0	15.4 ± 1.4	15.4	14.7	13.5	14.6	16.8	16.1	13.5–16.8	15.2 ± 1.2
ED	13.0	13.2	11.5	11.8	14.1	14.2	12.0	11.5–14.2	12.83 ± 1.1	13.0	12.7	11.9	11.8	13.3	13.0	11.8–13.3	12.6 ± 0.6
UEW	9.3	10.3	7.9	7.4	10.5	10.0	9.8	7.4–10.5	9.3 ± 1.2	8.8	9.5	9.4	9.5	10.3	9.2	8.8–10.3	9.5 ± 0.5
IND	10.2	10.6	8.3	7.7	11.2	10.5	10.3	7.7–11.2	9.8 ± 1.3	9.5	9.7	9.2	9.8	10.8	10.1	9.2–10.8	9.9 ± 0.6
IOD	7.4	7.5	6.2	6.3	7.5	8.0	8.1	6.2–8.1	7.3 ± 0.8	7.2	7.4	6.8	7.6	8.0	7.8	6.8–8.0	7.5 ± 0.4
DAE	16.2	17.4	13.6	13.5	17.5	17.0	16.8	13.5–17.5	16.0 ± 1.7	15.4	16.7	14.7	15.7	17.3	15.3	14.7–17.3	15.9 ± 0.9
DPE	28.1	28.8	23.3	24.7	30.0	28.5	26.0	23.3–30.0	27.1 ± 2.4	27.3	27.1	26.2	26.9	28.3	28.0	26.2–28.3	27.3 ± 0.8
NS	9.0	7.8	6.8	7.1	9.0	9.1	9.0	6.8–9.1	8.3 ± 1.0	8.2	7.5	6.4	7.3	9.0	9.1	6.4–9.1	7.9 ± 1.1
EN	6.9	7.0	6.5	6.6	7.2	6.7	7.1	6.5–7.2	6.9 ± 0.3	7.2	7.2	6.1	7.1	7.0	7.4	6.1–7.4	7.0 ± 0.5
TD	4.0	4.3	3.4	3.6	4.8	4.3	4.1	3.4–4.8	4.1 ± 0.5	4.4	3.6	4.2	4.3	4.4	4.8	3.6–4.8	4.3 ± 0.4
TYE	7.0	6.8	6.1	6.2	7.2	7.2	7.0	6.1–7.2	6.8 ± 0.5	6.3	6.8	6.5	7.5	7.1	6.8	6.3–7.5	6.8 ± 0.4
UAL	23.2	20.2	16.1	16.8	23.6	22.0	20.1	16.1–23.6	20.3 ± 2.9	18.4	18.6	15.6	17.6	22.0	19.0	15.6–22	18.5 ± 2.1
FAL	51.0	51.1	42.2	41.8	52.0	47.6	49.3	41.8–52.0	47.9 ± 4.3	44.5	42.8	42.4	41.5	51.4	46.8	41.5–51.4	44.9 ± 3.7
FeL	52.8	55.7	48.1	49.8	59.0	56.0	53.0	48.1–59.0	53.5 ± 3.8	53.7	53.2	47.9	52.6	59.0	51.2	47.9–59	52.9 ± 3.6
TbL	60.3	59.2	51.2	52.1	62.5	59.0	58.0	51.2–62.5	57.5 ± 4.2	57.8	56.0	52.4	54.7	61.0	56.8	52.4–61	56.5 ± 2.9
TbW	19.1	18.6	15.1	16.3	22.0	19.8	18.8	15.1–22.0	18.5 ± 2.3	17.5	17.3	16.5	18.5	22.0	19.0	16.5–22.0	18.5 ± 1.9
FoL	77.8	76.7	66.7	68.1	81.0	79.0	79.0	66.7–81.0	75.5 ± 5.7	75.2	72.1	68.4	69.3	79.3	74.6	68.4–79.3	73.2 ± 4.1
IMT	11.1	10.1	9.4	9.7	10.5	10.0	10.2	9.4–11.1	10.1 ± 0.6	10.3	9.9	10.6	9.2	10.2	10.5	9.2–10.6	10.1 ± 0.5
HL/SVL	0.37	0.37	0.40	0.40	0.37	0.38	0.39	0.37–0.4	0.38 ± 0.01	0.37	0.38	0.36	0.39	0.37	0.38	0.36–0.39	0.37 ± 0.01
HW/SVL	0.43	0.41	0.44	0.43	0.42	0.43	0.43	0.41–0.44	0.43 ± 0.01	0.43	0.43	0.41	0.43	0.42	0.42	0.41–0.43	0.42 ± 0.01
RL/SVL	0.16	0.14	0.15	0.16	0.16	0.16	0.16	0.14–0.16	0.16 ± 0.01	0.15	0.15	0.15	0.15	0.16	0.16	0.15–0.16	0.15 ± 0.00
HL/HW	0.86	0.89	0.91	0.92	0.88	0.88	0.89	0.86–0.92	0.89 ± 0.02	0.87	0.87	0.88	0.90	0.89	0.90	0.87–0.9	0.88 ± 0.01
ED/RL	0.81	0.89	0.86	0.86	0.83	0.85	0.74	0.74–0.89	0.83 ± 0.05	0.84	0.86	0.88	0.81	0.79	0.81	0.79–0.88	0.83 ± 0.04
TYE/TD	1.75	1.58	1.79	1.72	1.50	1.67	1.71	1.50–1.79	1.68 ± 0.10	1.43	1.89	1.55	1.74	1.61	1.42	1.42–1.89	1.61 ± 0.18
TD/ED	0.31	0.33	0.30	0.31	0.34	0.30	0.34	0.30–0.34	0.32 ± 0.02	0.34	0.28	0.35	0.36	0.33	0.37	0.28–0.37	0.34 ± 0.03
TbL/SVL	0.58	0.57	0.59	0.60	0.58	0.56	0.58	0.56–0.60	0.58 ± 0.01	0.58	0.57	0.57	0.56	0.57	0.55	0.55–0.58	0.57 ± 0.01
TbL/TbW	3.16	3.18	3.39	3.20	2.84	2.98	3.09	2.84–3.39	3.12 ± 0.17	3.30	3.24	3.18	2.96	2.77	2.99	2.77–3.3	3.07 ± 0.20

###### Ecological notes.

**Figure 12.**
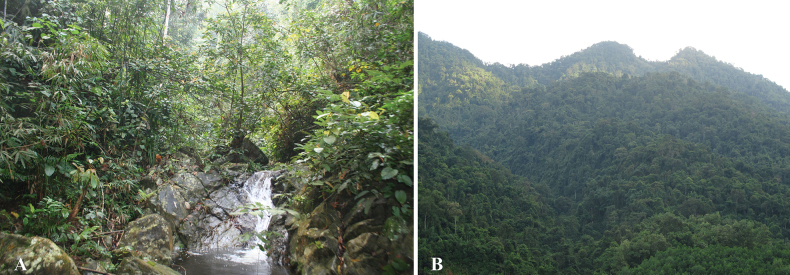
Habitat of *Quasipaaohlerae* sp. nov. in Xuan Lien Nature Reserve, Thanh Hoa Province, Viet Nam **A** microhabitat **B** evergreen forest.

Specimens were found between 19:00 and 23:00 in the headwaters of rocky streams (Fig. 12A). They were found in the water or on the ground of stream banks at elevations between 300 and 950 m a.s.l. The surrounding habitat was secondary forest of large, medium-sized, and small hardwoods mixed with shrubs and vines (Fig. 12B). Air temperatures at the sites ranged from 20.3–27.8 °C and relative humidity was 65–83%. Male advertisement calls and tadpoles of the species had not been recorded during our field surveys. Other amphibian species found at the sites included *Leptobrachellaeos* (Ohler, Wollenberg, Grosjean, Hendrix, Vences, Ziegler & Dubois, 2011), *Xenophryslancangica* Lyu, Wang & Wang, 2023, *Limnonectesbannaensis* Ye, Fei, Xie & Jiang, 2007, *Amolopstanfuilianae* Sheridan, Phimmachak, Sivongxay & Stuart, 2023, *Odorranachloronota* (Günther, 1876), *O.nasica* (Boulenger, 1903), *O.tiannanensis* (Yang & Li, 1980), *Hylaranamaosonensis* Bourret, 1937, *Kurixalus* sp., *Polypedatesmegacephalus* Hallowell, 1861, and *Rhacophorusorlovi* Ziegler & Köhler, 2001. During to field surveys in Vu Quang National Park (Ha Tinh Province) and Pu Hoat Nature Reserve (Nghe An Province) in April 2025, We also observed some individuals of the new species.

###### Distribution.

*Quasipaaohlerae* sp. nov. is currently known from Son La (Copia Nature Reserve), Thanh Hoa (Xuan Lien Nature Reserve), and Nghe An (Pu Hoat Nature Reserve) provinces, Vietnam. Data obtained from GenBank shows that this species was also recorded from Yunnan Province in China; Phongsaly Province in Laos; and Nan Province in Thailand (Suwannapoom et al. 2021; see Discussion below).

###### Comparisons.

We compared the new species with its congeners. *Quasipaaohlerae* sp. nov. differs from *Q.verrucospinosa* by having nuptial spines on all fingers of males (vs absent on finger IV); males with nuptial spines on ventral surface of arms (vs absent); dense spines on lower lip and throat of males (vs small and scattered); a smaller ratio of TD/ED (0.32, *n* = 7 in males and 0.34, *n* = 6 in females vs 0.53, *n* = 7 in males and 0.50, *n* = 7 in females); a greater ratio of TYE/TD (1.68, *n* = 7 in males and 1.61, *n* = 6 in females vs 1.16, *n* = 7 in males and 1.14, *n* = 7 in females), inner metatarsal tubercle oval (vs inner metatarsal tubercle round); different dorsal color pattern (dark brown vs yellowish grey); different ventral color pattern (immaculate white vs pale yellow); iris dark green (vs pale copper); and females with wholly unpigmented eggs (vs melanic poles).

*Quasipaaohlerae* sp. nov. differs from *Q.acanthophora* by having the dorsum with thick ridges (vs small tubercles); males with nuptial spines on ventral surface of arms (vs absent); males with nuptial spines on all fingers (vs absent on finger IV); dense spines present on throat and chest of males (vs small and scattered); and iris dark green (vs copper on upper and greyish on lower).

*Quasipaaohlerae* sp. nov. differs from *Q.boulengeri* by having the dorsum with thick ridges and round tubercles (vs elongate ridges), males with nuptial spines on all fingers (vs absent on finger IV), males with nuptial spines on throat and ventral surface of arms (vs absent), different ventral color pattern (immaculate white vs pale yellow), and iris dark green (vs copper on upper and greyish on lower).

*Quasipaaohlerae* sp. nov. differs from *Q.courtoisi* by having a smaller size in males (SVL 86.7–107.8 mm, *n* = 7 vs 126 mm, *n* = 1), males with nuptial spines on throat and ventral surface of arms (vs absent) and males with nuptial spines on all fingers (vs absent on finger IV).

**Figure 13.**
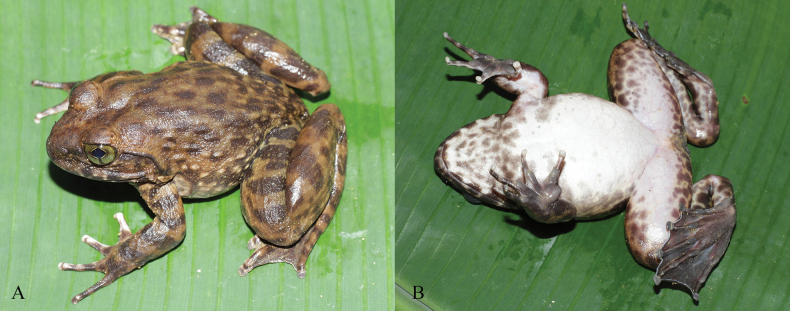
*Quasipaadelacouri* in life **A** dorsolateral view **B** ventral view (IEBR A.5017, male).

**Figure 14.**
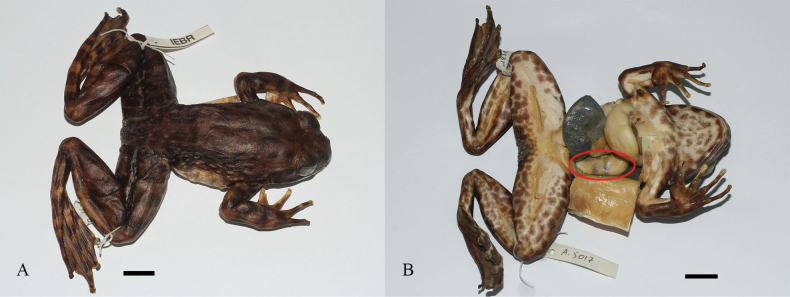
*Quasipaadelacouri* (IEBR A.5017, male) in preservative **A** dorsolateral view **B** ventral view. The red oval indicates testicles of male. Scale bars: 10 mm

**Figure 15.**
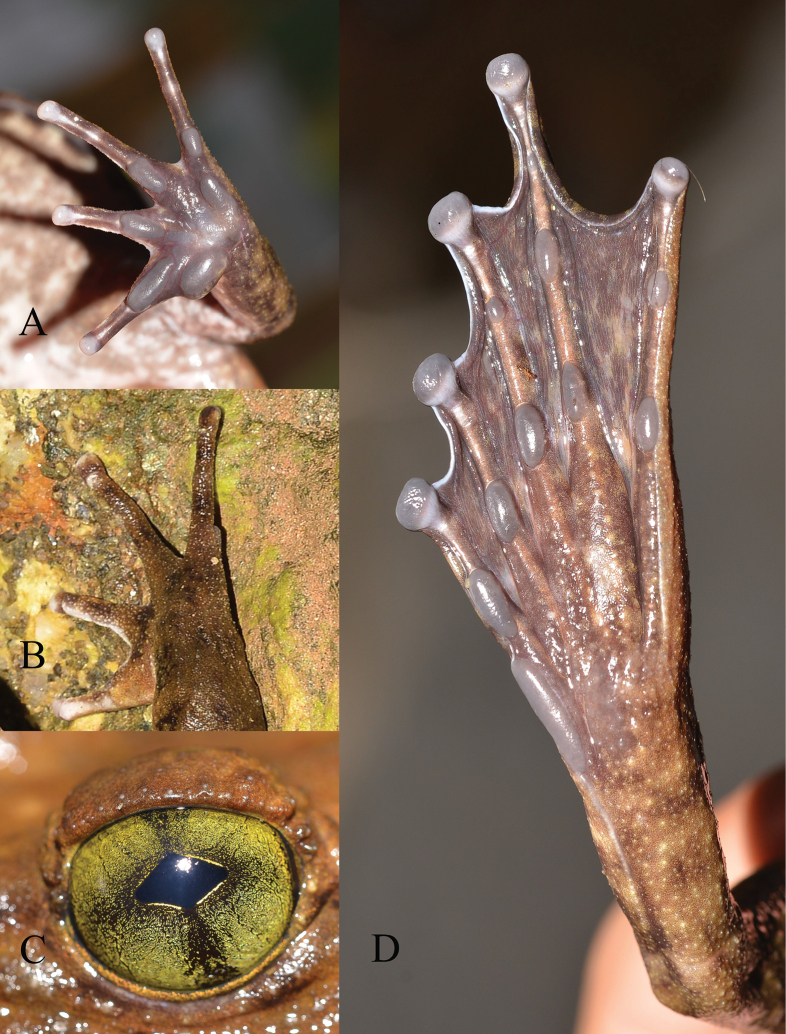
*Quasipaadelacouri***A** lower left hand of male (IEBR A.5017) **B** upper right hand of male (IEBR A.5017) **C** iris of male (IEBR A.5017) **D** lower right foot of male (IEBR A.5017).

*Quasipaaohlerae* sp. nov. differs from *Q.delacouri* by having the dorsum with thick ridges and round tubercles (vs smooth); males with nuptial pad on fingers I and II (vs absent); and males with nuptial spines (vs absent); different dorsal color pattern (dark brown vs brick red with black spots); and tibio-tarsal articulation reaching to nostril (vs tibio-tarsal articulation reaching to tip of snout), a greater ratio of TD/ED (0.32, *n* = 7 in males and 0.34, *n* = 6 in females vs 0.26, *n* = 3 in males and 0.24, *n* = 3 in females); a smaller ratio of TYE/TD (1.68, *n* = 7 in males and 1.61, *n* = 6 in females vs 2.15, *n* = 3 in males and 1.93, *n* = 3 in females) (Figs 13–15, Table 5).

*Quasipaaohlerae* sp. nov. differs from *Q.exilispinosa* by having a larger size (SVL 86.7–107.8 mm, *n* = 7 in males and 92.7–107.0 mm, *n* = 6 in females vs SVL 44.2–66.5 mm, *n* = 20 in males and 40.0–63.3 mm, *n* = 20 in females); dorsum with thick ridges (vs small tubercles); males with nuptial spines on throat and ventral surface of arms (vs absent); males with nuptial spines on all fingers (vs absent on finger IV); and iris dark green (vs copper on upper and greyish on lower).

*Quasipaaohlerae* sp. nov. differs from *Q.fasciculispina* by having a smaller ratio of TYE/TD in males (1.68, *n* = 7 vs 2.0, *n* = 1); each chest tubercle with only one black spine in males (vs each chest tubercle with 5–10 black spines); iris dark green (vs bright copper-colored); and external vocal sac absent (vs vocal sac openings in floor of mouth).

*Quasipaaohlerae* sp. nov. differs from *Q.jiulongensis* by having the dorsum with thick ridges (vs small tubercles); males with nuptial spines on throat and ventral surface of arms of males (vs absent); males with nuptial spines on all fingers (vs absent on fingers III and IV); the absence of pale-colored longitudinal stripes on upper jaw edge (vs present); and the absence of four or five yellow dorsal dots arranged in longitudinal rows (vs present).

*Quasipaaohlerae* sp. nov. differs from *Q.robertingeri* by having the dorsum with thick ridges and round tubercles (vs elongate ridges); males with nuptial spines on all fingers (vs absent on finger IV); males with nuptial spines on throat and ventral surface of arms (vs absent); different ventral color pattern (immaculate white vs pale yellow); and iris dark green (vs copper on upper and greyish on lower).

*Quasipaaohlerae* sp. nov. differs from *Q.shini* by the males having nuptial spines on all fingers (vs absent on finger IV), on throat and ventral surface of arms (vs absent), and each chest tubercle with only one black spine in males (vs each chest tubercle with 3–8 black spines).

*Quasipaaohlerae* sp. nov. differs from *Q.spinosa* by having the dorsum with thick ridges (vs small tubercles); the absence of pale-colored longitudinal stripes on upper jaw edge (vs present); males with nuptial spines on throat and ventral surface of arms of males (vs absent); and males with nuptial spines on all fingers (vs absent on fingers III and IV).

*Quasipaaohlerae* sp. nov. differs from *Q.taoi* by its lager size (SVL 86.7–107.8 mm, *n* = 7 in males and 92.7–107.0 mm, *n* = 6 in females vs 79.6–84.3 mm, *n* = 3 in males and 64.6–69.9 mm, *n* = 3 in females); a greater ratio of TYE/TD (1.68, *n* = 7 in males and 1.61, *n* = 6 in females vs 1.11, *n* = 3 in males and 1.20, *n* = 3 in females); the presence of nuptial spines on chest and belly in males (vs absent); iris dark green (vs dark copper); and tibio-tarsal articulation reaching to nostril (vs tibio-tarsal articulation reaching to tip of snout).

*Quasipaaohlerae* sp. nov. differs from *Q.yei* by its larger size (SVL 86.7–107.8 mm, *n* = 7 in males and 92.7–107.0 mm, *n* = 6 in females vs 49.7–64.0 mm, *n* = 25 in males and 69.0–83.0 mm, *n* = 25 in females); males with nuptial spines on lower flanks, ventral surface of forelimbs, lower lip, throat, chest, 2/3 anterior part of belly (vs absent); males with nuptial spines on all fingers (vs absent); and the absence of nuptial spines around vent (vs present).

###### Etymology.

The new species is named in honor of our colleague and friend, Prof. Dr. Annemarie Ohler from the Département de Systématique et Evolution, Muséum National d’Histoire Naturelle, Paris, France, in recognition of her great contributions towards a better understanding of the amphibian systematics of the Indochinese region. We recommend “Ohler’s Spiny Frog” as the common English name of the new species and the common name in Vietnamese as “Ếch gai sần ohler”.

##### ﻿ Quasipaa binhi sp. nov.

Taxon classificationAnimaliaAnuraDicroglossidae

0C443FD8-DB6F-5ADB-8080-B11DBD3A9B6C

https://zoobank.org/9AA69B06-3686-4DAF-9A2F-145A89FE0D52

**Figure 16.**
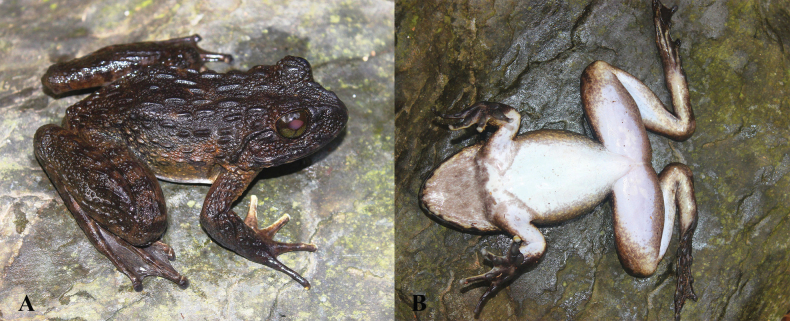
*Quasipaabinhi* sp. nov., holotype (IEBR A.5174, male) in life **A** dorsolateral view **B** ventral view.

**Figure 17.**
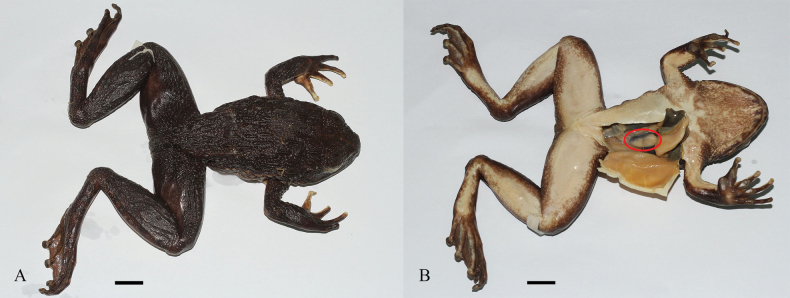
*Quasipaabinhi* sp. nov., holotype (IEBR A.5174, male) in preservative **A** dorsolateral view **B** ventral view. The red oval indicates testicles of male. Scale bars: 10 mm

**Figure 18.**
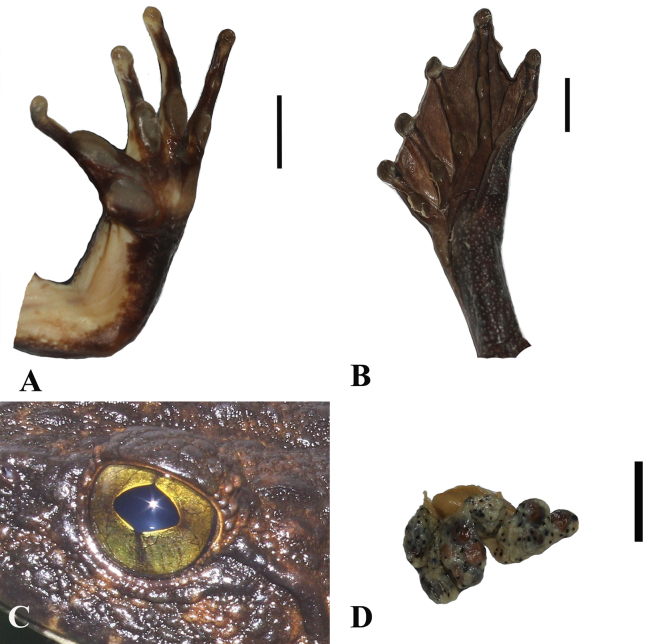
*Quasipaabinhi* sp. nov. **A** lower left hand of holotype (IEBR A.5174, male) **B** lower right foot of holotype (IEBR A.5174, male) **C** iris of holotype (IEBR A.5174, male) **D** eggs of paratype (IEBR A.5180, female). Scale bars: 10 mm

**Figure 19.**
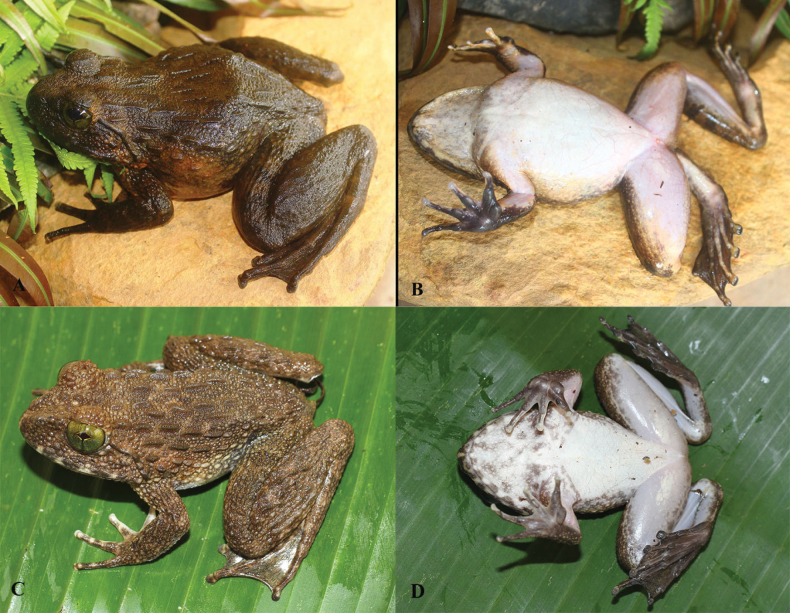
*Quasipaabinhi* sp. nov., paratypes (IEBR A.5180, female) in life **A** dorsolateral view (IEBR A.5180, female) **B** ventral view (IEBR A.5180, female) **C** dorsolateral view (IEBR A.5183, male) **D** ventral view (IEBR A.5183, male).

Figs 16 , 17 , 18 , 19 , Table 7

Quasipaa delacouri : Yan et al. 2021: 1–7.
Quasipaa cf. verrucospinosa 2: Suwannapoom et al. 2021: 1–12.

###### Material examined.

***Holotype*.** • IEBR A.5174, adult male, collected by T. Q. Nguyen and C. T. Pham, on 11 March 2015, in Dong Chau-Khe Nuoc Trong Nature Reserve (16°56.461'N, 106°38.299'E, at an elevation of 447 m a.s.l.), Le Thuy District, Quang Binh Province, Vietnam. ***Paratypes*.** (*n* = 8) • IEBR A.5181, adult female, the same collection data as for holotype; IEBR A.5178, 5179, two adult males and IEBR A.5175, 5180, two adult females, collected by T. Q. Nguyen and C. T. Pham, on 18 March 2015, in Dong Chau - Khe Nuoc Trong Nature Reserve (16°59.273'N, 106°36.568'E, at an elevation of 382 m a.s.l.), Le Thuy District, Quang Binh Province, Vietnam; • IEBR A.5182, adult male, collected by C. T. Pham and C. V. Hoang, on 27 July 2015, in Dong Chau-Khe Nuoc Trong Nature Reserve (16°57.036'N, 106°37.504'E, at an elevation of 300 m a.s.l.), Le Thuy District, Quang Binh Province, Vietnam; • IEBR A.5183, adult male and IEBR A.5184, adult female, collected by C. T. Pham and T. V. Nguyen, on 5 June 2017, in Sao La Nature Reserve (16°04.306'N, 107°29.062'E, at an elevation of 750 m a.s.l.), A Luoi District, Thua Thien Hue Province, Vietnam.

###### Diagnosis.

Both morphological characteristics (body very stout, skin rough with dermal ridges and tubercles, forelimbs of males strongly enlarged, with inner side of arms or fingers or chest and belly with black spines) (Fei et al. 2009) and molecular data revealed the new species to be nested within *Quasipaa*. *Quasipaabinhi* sp. nov. is distinguishable from its congeners by a combination of the following morphological characteristics: (1) SVL 76.9–101.1 mm in males and 88.5–123.4 mm in females; (2) head broader than long (HL/HW 0.96 in males, 0.96 in females); (3) vomerine teeth present; (4) external vocal sacs absent; (5) tympanum visible, round; (6) dorsum with thin and elongate ridges intermixed with small round tubercles; (7) flanks covered by oval and round tubercles; (8) supratympanic fold present; (9) dorsolateral fold absent; (10) dorsum and dorsal surface of fore- and hindlimbs with small black spines, scattered; (11) nuptial pad absent on finger I in males (12) ventral surface of body and all fingers without spines in males; (13) eggs yellowish cream with melanic poles in females; (14) toes fully webbed to distal end of terminal phalanx; and (15) in life, dorsum dark brown and belly immaculate white, and iris dark green.

###### Description of holotype.

A large frog (SVL 101.1 mm); habitus robust with enlarged head (HL/SVL 0.40, HW/SVL 0.42); head broader than long (HL 40.6 mm, HW 42.0 mm); snout round anteriorly in dorsal view, projecting beyond lower jaw; nostril lateral, closer to eye than to the tip of snout (NS 8.3 mm, EN 7.4 mm); canthus rostralis indistinct; loreal region oblique and slightly concave; rostral length greater than eye diameter (RL 15.8 mm, ED 12.2 mm); interorbital distance smaller than internarial distance and upper eyelid width (IOD 6.7 mm, IND 9.3 mm, UEW 9.9 mm); tympanum slightly visible (TD 4.4 mm) smaller than the distance from tympanum to eye (TYE 6.1 mm), ~ 36% eye diameter; vomerine teeth in two oblique ridges; tongue cordiform, notched posteriorly; external vocal sac absent.

***Forelimbs***: arms short; upper arm length (UAL) 17.2 mm, forearm length (FAL) 45.1 mm; relative finger lengths: II<I<IV<III; fingers free of webbing; narrow dermal ridge on sides of fingers present on fingers II, III; tips of fingers swollen, not expanded; subarticular tubercles prominent, round, formula 1, 1, 2, 2; inner metatarsal tubercle oval; outer metatarsal tubercle elongate; nuptial pad absent.

***Hindlimbs***: tibia length longer than thigh length (FeL 56.0 mm, TbL 61.0 mm), ~ 3.7× longer than wide (TbW 16.2 mm); tips of toes swollen, round; relative length of toes: I<II<V<III<IV; toes fully webbed to distal end of terminal phalanx; dermal ridge present on outer sides of toes I and V; subarticular tubercles prominent, elongate, formula 1, 1, 2, 3, 2; inner metatarsal tubercle elongate; outer metatarsal tubercle absent; tibio-tarsal articulation reaching to tip of snout.

***Skin texture in life***: dorsal surface of head with oval and round tubercles, dorsum with thin and elongate ridges intermixed with small round tubercles; flanks covered by oval and round tubercles; supratympanic fold distinct, extending from eye to angle of jaw; dorsolateral fold absent; dorsal surface of forelimb and hindlimb with thin and elongate ridges intermixed with small tubercles; belly and ventral surface of thigh smooth.

***Nuptial spines***: dorsum, upper flanks, upper lip, and dorsal surface of fore- and hindlimbs with small spines, scattered; ventral surface of body and fingers without spines.

***Coloration in life***: iris dark green; dorsum and upper part of flanks dark brown; lower part of flanks yellow brown with white tubercles and black spines on top; dorsal surface of limbs yellowish brown with dark crossbars; throat and chest white with brown markings; ventral surface of limbs and belly immaculate white; toe webbing dark brown.

***Coloration in preservative***: coloration in preservative is the same in life but somewhat faded.

###### Sexual dimorphism.

Measurements and morphological characters of the type series are provided in Table 7. The male specimens have spines on dorsum, upper flanks, upper lip, and dorsal surface of fore- and hindlimbs small, scattered. The females contained yellowish cream eggs with melanic poles.

**Table 7.** Measurements (in mm) and proportions of the type series of *Quasipaabinhi* sp. nov. (H = holotype, P = paratype, SD = standard deviation, M = male, F = female; for other abbreviations see Materials and methods).

Voucher	IEBR A.5174	IEBR A.5179	IEBR A.5182	IEBR A.5178	IEBR A.5183	Min–Max	Mean ± SD	IEBR A.5175	IEBR A.5181	IEBR A.5180	IEBR A.5184	Min–Max	Mean ± SD
Sex	M	M	M	M	M	(*n* = 5)	(*n* = 5)	F	F	F	F	(*n* = 4)	(*n* = 4)
Type status	H	P	P	P	P			P	P	P	P		
SVL	101.1	80.4	92.9	78.1	76.9	76.9–101.1	85.9 ± 10.6	123.4	107.4	88.5	115.1	88.5–123.4	108.6 ± 14.9
HL	40.6	31.0	35.9	32.3	30.0	30.0–40.6	34.0 ± 4.3	47.2	42.7	36.3	43.4	36.3–47.2	42.4 ± 4.5
HW	42.0	32.9	38.2	33.5	31.1	31.1–42	35.6 ± 4.5	49.1	45.0	37.0	46.3	37.0–49.1	44.3 ± 5.2
MN	33.8	28.5	29.8	27.1	26.3	26.3–33.8	29.1 ± 3.0	39.5	35.4	31.0	38.2	31.0–39.5	36.0 ± 3.8
MFE	27.0	23.0	25.0	21.6	21.9	21.6–27	23.7 ± 2.3	32.3	28.3	24.8	32.6	24.8–32.6	29.5 ± 3.7
MBE	16.8	14.3	15.0	13.4	14.0	13.4–16.8	14.7 ± 1.3	22.1	17.4	15.6	20.1	15.6–22.1	18.8 ± 2.9
RL	15.8	13.0	14.4	12.1	13.7	12.1–15.8	13.8 ± 1.4	17.2	16.7	13.7	18.1	13.7–18.1	16.4 ± 1.9
ED	12.2	10.6	11.6	10.4	9.8	9.8–12.2	10.9 ± 1.0	12.6	13.2	10.5	13.4	10.5–13.4	12.4 ± 1.3
UEW	9.9	8.7	9.2	8.5	7.4	7.4–9.9	8.7 ± 0.9	12.3	10.1	9.6	11.5	9.6–12.3	10.9 ± 1.2
IND	9.3	7.6	8.6	7.6	7.5	7.5–9.3	8.1 ± 0.8	10.7	9.9	8.5	11.2	8.5–11.2	10.1 ± 1.2
IOD	6.7	6.4	6.5	5.7	6.1	5.7–6.7	6.3 ± 0.4	8.5	7.3	4.8	8.1	4.8–8.5	7.2 ± 1.7
DAE	17.7	14.4	15.8	12.6	13.6	12.6–17.7	14.8 ± 2.0	20	17.6	15.7	18.6	15.7–20	18 ± 1.8
DPE	28.0	22.2	25.6	24.4	20.9	20.9–28	24.2 ± 2.8	31.6	27.6	25.4	29.5	25.4–31.6	28.5 ± 2.6
NS	8.3	6.8	7.6	6.2	7.4	6.2–8.3	7.3 ± 0.8	9.2	8.8	7.5	9.8	7.5–9.8	8.8 ± 1.0
EN	7.4	6.2	6.8	5.9	6.3	5.9–7.4	6.5 ± 0.6	8.1	7.9	6.2	8.3	6.2–8.3	7.6 ± 0.9
TD	4.4	3.6	4.5	4.0	3.8	3.6–4.5	4.0 ± 0.4	5.0	5.3	4.3	4.4	4.3–5.3	4.8 ± 0.5
TYE	6.1	4.5	5.5	4.2	4.4	4.2–6.1	4.9 ± 0.8	8.8	7.9	6.7	7.8	6.7–8.8	7.8 ± 0.8
UAL	17.2	14.3	16.5	13.5	13.9	13.5–17.2	15.1 ± 1.7	19.2	19.9	14.9	20.2	14.9–20.2	18.5 ± 2.5
FAL	45.1	35.7	39.0	35.3	33.3	33.3–45.1	37.7 ± 4.6	53.5	48.9	39.3	50	39.3–53.5	47.9 ± 6.1
FeL	56.0	40.1	48.9	39.2	41.5	39.2–56	45.1 ± 7.2	66.3	56	49.1	61.9	49.1–66.3	58.3 ± 7.4
TbL	61.0	43.5	51.2	43.8	42.6	42.6–61	48.4 ± 7.8	67.6	60.4	50	62.4	50–67.6	60.1 ± 7.4
TbW	16.6	11.0	16.1	12.7	12.8	11–16.6	13.8 ± 2.4	20	16.5	14.4	19.6	14.4–20	17.6 ± 2.7
FoL	75.2	58.9	64.4	58.8	52.4	52.4–75.2	61.9 ± 8.5	90.1	80.2	67.3	86.4	67.3–90.1	81 ± 10.0
IMT	9.8	6.8	8.0	6.8	6.6	6.6–9.8	7.6 ± 1.3	10.9	9.0	6.2	10.7	6.2–10.9	9.2 ± 2.2
HL/SVL	0.40	0.39	0.39	0.41	0.39	0.39–0.4	0.40 ± 0.01	0.38	0.4	0.41	0.38	0.41–0.38	0.39 ± 0.01
HW/SVL	0.42	0.41	0.41	0.43	0.40	0.40–0.42	0.41 ± 0.01	0.4	0.42	0.42	0.4	0.42–0.4	0.41 ± 0.01
RL/SVL	0.16	0.16	0.15	0.16	0.18	0.16–0.16	0.16 ± 0.01	0.14	0.16	0.15	0.16	0.15–0.15	0.15 ± 0.01
HL/HW	0.97	0.94	0.94	0.96	0.96	0.96–0.97	0.96 ± 0.01	0.96	0.95	0.98	0.94	0.98–0.96	0.96 ± 0.02
ED/RL	0.78	0.81	0.81	0.86	0.71	0.81–0.78	0.79 ± 0.05	0.73	0.79	0.77	0.74	0.77–0.74	0.76 ± 0.03
TYE/TD	1.38	1.25	1.22	1.05	1.18	1.17–1.36	1.22 ± 0.12	1.75	1.48	1.56	1.77	1.56–1.64	1.64 ± 0.14
TD/ED	0.36	0.34	0.38	0.38	0.38	0.34–0.38	0.37 ± 0.02	0.4	0.4	0.41	0.33	0.33–0.41	0.38 ± 0.04
TbL/SVL	0.60	0.54	0.55	0.56	0.55	0.55–0.60	0.56 ± 0.02	0.55	0.56	0.56	0.54	0.56–0.55	0.55 ± 0.01
TbL/TbW	3.67	3.97	3.18	3.44	3.33	3.88–3.67	3.52 ± 0.31	3.38	3.65	3.47	3.18	3.47–3.38	3.41 ± 0.2

###### Ecological notes.

**Figure 20.**
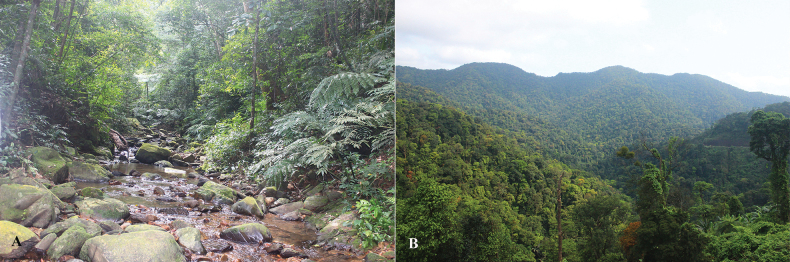
Habitat of *Quasipaabinhi* sp. nov. in Dong Chau-Khe Nuoc Trong Nature Reserve, Quang Binh Province, Viet Nam **A** microhabitat **B** evergreen forest.

Specimens were found between 19:00 and 23:00 in the headwaters of rocky streams (Fig. 20A). They were found in the water or on the ground of stream banks at elevations between 300 and 750 m a.s.l. The surrounding habitat was secondary forest of large, medium-sized, and small hardwoods mixed with shrubs and vines (Fig. 20B). Air temperatures at the sites ranged from 20.1–25.7 °C and relative humidity was 83–95%. Male advertisement calls and tadpoles of the species had not been recorded during our field surveys. Other amphibian species found at the sites included *Leptobrachiumchapaense* (Bourret, 1937), *Xenophrystruongsonensis* Luong, Hoang, Pham, Nguyen, Orlov, Ziegler & Nguyen, 2022, *Limnonecteskiziriani* Pham, Le, Ngo, Ziegler & Nguyen, 2018, *L.poilani* (Bourret, 1942), *Amolopscompotrix* (Bain, Stuart & Orlov, 2006), *Papuranaattigua* (Inger, Orlov & Darevsky, 1999); *Odorranagigatympana* (Orlov, Ananjeva & Ho, 2006), *O.khalam* (Stuart, Orlov & Chan-ard, 2005), *Hylaranamaosonensis* Bourret, 1937, and *Rhacophorusorlovi* Ziegler & Köhler, 2001.

###### Distribution.

*Quasipaabinhi* sp. nov. is currently known from Quang Binh (Dong Chau-Khe Nuoc Trong Nature Reserve) and Thua Thien Hue (Sao La Nature Reserve) provinces, Vietnam. Data obtained from GenBank shows that this species was also recorded from Xekong Province, Laos (Suwannapoom et al. 2021; see Discussion below).

###### Comparisons.

We compared the new species with its congeners. *Quasipaabinhi* sp. nov. differs from *Q.ohlerae* sp. nov. by having the dorsum with thin and elongate ridges (vs with thick ridges); the absence nuptial spines on all fingers and ventral surface of forelimbs in males (vs present); the absence spines on lower flanks, ventral surface of forelimbs, lower lip, throat, chest, 2/3 anterior part of belly in males (vs present); and females with melanic pole eggs (vs wholly unpigmented); and a smaller ratio of TYE/TD in males (1.22, *n* = 5 vs 1.68, *n* = 7).

*Quasipaabinhi* sp. nov. differs from *Q.verrucospinosa* by the absence nuptial spines on all fingers in males (vs present of nuptial spines on fingers I, II, III); dorsum with thin and elongate ridges (vs dorsum with thick ridges); a smaller ratio of TD/ED (0.37, *n* = 5 in males and 0.38, *n* = 4 in females vs 0.53, *n* = 7 in males and 0.50, *n* = 7 in females); a greater ratio of TYE/TD (1.22, *n* = 5 in males and 1.64, *n* = 64 in females vs 1.16, *n* = 7 in males and 1.14, *n* = 7 in females); inner metatarsal tubercle oval (vs inner metatarsal tubercle round); different dorsal color pattern (dark brown vs yellowish grey); different ventral color pattern (immaculate white vs pale yellow); and iris dark green (vs pale copper).

*Quasipaabinhi* sp. nov. differs from *Q.acanthophora* by having the dorsum with thin and elongate ridges (vs small tubercles); the absence nuptial spines on all fingers in males (vs present on fingers I, II, III); the absence of spines on throat and chest in males (vs present); and iris dark green (vs copper on upper and greyish on lower).

*Quasipaabinhi* sp. nov. differs from *Q.boulengeri* by the absence nuptial spines on all fingers in males (vs present on fingers I, II, III); the absence of spines on chest and belly in males (vs present); different ventral color pattern (immaculate white vs pale yellow); and iris dark green (vs copper on upper and greyish on lower).

*Quasipaa bìnhi* sp. nov. differs from *Q.courtoisi* by having a smaller size in males (SVL 76.9–101.1 mm, *n* = 5 *vs* 126 mm, *n* = 1); the absence of spines on chest in males (*vs* present); and the absence nuptial spines on all fingers in males (vs present of nuptial spines on fingers I, II, III).

*Quasipaabinhi* sp. nov. differs from *Q.delacouri* by having the dorsum with thin and elongate ridges (vs smooth); dorsal surface of forelimbs and hindlimbs with thin and elongate ridges intermixed with small tubercles (vs smooth); different dorsal color pattern (dark brown vs brick red with black spots); and dorsum, upper flanks, upper lip, and dorsal surface of fore- and hindlimbs with small spines, scattered in males (vs absent); a greater ratio of TD/ED (0.37, *n* = 4 in males and 0.38, *n* = 4 in females vs 0.26, *n* = 3 in males and 0.24, *n* = 3 in females); a smaller ratio of TYE/TD (1.22, *n* = 5 in males and 1.64, *n* = 4 in females vs 2.15, *n* = 3 in males and 1.93, *n* = 3 in females) (Figs 13–15, Table 5).

*Quasipaabinhi* sp. nov. differs from *Q.exilispinosa* by having a larger size in males (SVL 76.9–101.1 mm, *n* = 5 in males and 88.5–123.4 mm, *n* = 4 in females vs SVL 44.2–66.5 mm, *n* = 20 in males and 40.0–63.3 mm, *n* = 20 in females); the dorsum with thin and elongate ridges (vs small tubercles); the absence of spines on chest of males (vs present); the absence nuptial spines on all fingers in males (vs present on fingers I, II, III); different ventral color pattern (immaculate white vs pale yellow); and iris dark green (vs copper on upper and greyish on lower).

*Quasipaabinhi* sp. nov. differs from *Q.fasciculispina* by having a smaller ratio of TYE/TD in males (1.22, *n* = 5 vs 2.0, *n* = 1); the absence of spines on chest in males (*vs* each chest tubercle with 5–10 black spines); the absence nuptial spines on all fingers in males (vs present of nuptial spines on fingers I, II, III); iris dark green (vs bright copper-colored); and external vocal sac absent (vs vocal sac openings in floor of mouth).

*Quasipaabinhi* sp. nov. differs from *Q.jiulongensis* by having the dorsum with thin and elongate ridges (vs small tubercles); the absence of spines on chest of males (vs present); the absence nuptial spines on all fingers in males (vs present on fingers I and II); the absence of pale-colored longitudinal stripes on upper jaw edge (vs present); and the absence of 4 or 5 yellow dorsal dots arranged in longitudinal rows (vs present).

*Quasipaabinhi* sp. nov. differs from *Q.robertingeri* by having different dorsal pattern (dark brown vs pale yellowish grey); the absence of spines on chest of males (vs present); the absence nuptial spines on all fingers in males (vs present of nuptial spines on fingers I, II, III); the absence of spines on chest and belly of males (vs present); and iris dark green (vs copper on upper and greyish on lower).

*Quasipaabinhi* sp. nov. differs from *Q.shini* by having the dorsum with thin and elongate ridges (vs dorsum with thick ridges); the absence nuptial spines on all fingers in males (vs present on fingers I, II, III); and the absence of spines on chest of males (vs each chest tubercle with 3–8 black spines).

*Quasipaabinhi* sp. nov. differs from *Q.spinosa* by having the dorsum with thin and elongate ridges and round tubercles (vs small tubercles); the absence of pale-colored longitudinal stripes on upper jaw edge (vs present); the absence nuptial spines on all fingers in males (vs present on fingers I and II); and the absence of spines on chest of males (vs present).

*Quasipaabinhi* sp. nov. differs from *Q.taoi* by its larger size in females (88.5–123.4 mm, *n* = 4, in females vs 64.6–69.9 mm, *n* = 3 in females); the dorsum with thin and elongate ridges (vs dorsum with thick ridges); the absence nuptial spines on all fingers and ventral surface of forelimbs in males (vs present); and iris dark green (vs dark copper).

*Quasipaabinhi* sp. nov. differs from *Q.yei* by its larger size in males (SVL 76.8–101.1 mm, *n* = 5 in males and 88.5–123.4 mm, *n* = 4 in females vs 49.7–64.0 mm, *n* = 25 in males and 69.0–83.0 mm, *n* = 25 in females); dorsum with thin and elongate ridges (vs small tubercles); different dorsal pattern (dark brown vs pale yellowish brown); and the absence of nuptial spines around and inside vent (vs present).

###### Etymology.

The new species is named in honor of our colleague and friend, late Assoc. Prof. Dr. Binh Van Nguyen from the Hue University, Hue City, Vietnam, in recognition of his contributions on ecological research of amphibians in Vietnam. We recommend “Binh’s Spiny Frog” as the common English name of the new species and the common name in Vietnamese as “Ếch gai sần bình”.

## ﻿Discussion

*Quasipaaverrucospinosa* was originally described from Sapa (Lao Cai Province) and Tam Dao National Park (Vinh Phuc Province), northern Vietnam (Bourret 1942). Suwannapoom et al. (2021) assigned the populations from Nghe An (Vietnam), Laos, China, and Thailand to *Q.verrucospinosa* sensu stricto. The authors identified several populations with unconfirmed status: Q.cf.verrucospinosa 1 from Tam Dao, Vinh Phuc Province in Vietnam; Q.cf.verrucospinosa 2 from Xe Kong Province in Laos; Q.cf.verrucospinosa 3 from Ngoc Linh, Kon Tum Province in Vietnam and Xe Kong Province in Laos (Suwannapoom et al. 2021). The population of Q.cf.verrucospinosa 3 was recently described as *Q.taoi* by Pham et al. (2022). Based on our results, Q.cf.verrucospinosa 1 (Suwannapoom et al. 2021), including populations from the type locality in Hoang Lien National Park (Sa Pa District, Lao Cai Province) and from Tam Dao National Park (Vinh Phuc Province) and other localities in the North, namely Bac Me Nature Reserve (Ha Giang Province), Cham Chu Nature Reserve and Na Hang District (Tuyen Quang Province), should be assigned to *Q.verrucospinosa* sensu stricto; the populations from Son La, Thanh Hoa, Nghe An, and Ha Tinh provinces (Vietnam), Yunnan Province (China), Phongsaly Province (Laos), and Nan Province (Thailand), are herein re-identified as *Quasipaaohlerae* sp. nov.; and Q.cf.verrucospinosa 2 (Suwannapoom et al. 2021), including populations from Thua Thien Hue and Quang Binh provinces in Central Vietnam and Xe Kong Province of Laos should be *Quasipaabinhi* sp. nov. In addition, previous records of *Q.delacouri* from Xe Kong Province (Laos), Da Nang, and Thua Thien Hue provinces (Vietnam) by Yan et al. (2021) are re-identified as *Quasipaabinhi* sp. nov. The distribution of *Q.delacouri* is restricted to northern Vietnam.

Furthermore, this study also showed that *Q.spinosa* (Suwannapoom et al. 2021) or *Quasipaa* sp.2 (Yan et al. 2021), including the populations from Phu Tho Province (Vietnam) and Yunnan Province (China) represent an unnamed species. *Quasipaaspinosa* from Guangxi Province (Chongzuo, Dongzhong, Fulong, and Yuanbaoshan), China formed a distinctive lineage M3 following Yan et al. (2021) and *Q.acanthophora* from Lang Son Province (Vietnam) in this study are in the same clade. However, because of the lack of voucher specimens for morphological comparisons, we provisionally consider the lineage M3 from Guangxi Province as a new record of *Q.acanthophora* from China. Further studies are needed to explore the actual diversity of the genus *Quasipaa*, particularly in the border regions between Vietnam and China as well as between Vietnam and Laos.

## Supplementary Material

XML Treatment for Quasipaa verrucospinosa

XML Treatment for Quasipaa ohlerae

XML Treatment for Quasipaa binhi

## ﻿Additional information

### Author ORCIDs

Cuong The Pham https://orcid.org/0000-0001-5158-4526

Chung Van Hoang https://orcid.org/0000-0002-0709-974X

Tien Quang Phan https://orcid.org/0000-0002-2738-5364

Anh Van Pham https://orcid.org/0000-0002-6023-3418

An Vinh Ong https://orcid.org/0000-0003-3683-3832

Vien Hong Thi Nguyen https://orcid.org/0000-0002-3744-3054

Thomas Ziegler https://orcid.org/0000-0002-4797-609X

Truong Quang Nguyen https://orcid.org/0000-0002-6601-0880

### Data availability

All of the data that support the findings of this study are available in the main text.

## ﻿Appendix 1

**Specimens examined**

*Quasipaaacanthophora* (*n* = 11): Vietnam: Lang Son Province: Cao Loc District: Mau Son Commune: IEBR A.5030–5032 (males) and IEBR A.5033–5035 (females); Vietnam: Bac Giang Province: Son Dong District: Tay Yen Tu Nature Reserve: IEBR A.2013.62 (male), IEBR A.2013.63 and 2013.64 (females), IEBR 3625–3626 (males).

*Quasipaaboulengeri* (*n* = 13): Vietnam: Cao Bang Province: Nguyen Binh District: Phia Oac-Phia Den National Park: IEBR A.5007–5009 (males), IEBR A.5010–5014 (females), IEBR A.5039–5041 (males); Vietnam: Ha Giang Province: Quan Ba District: IEBR A.5015–5016 (males).

*Quasipaadelacouri* (*n* = 6): Vietnam: Tuyen Quang Province: Cham Chu Nature Reserve: IEBR A.5017 and IEBR A.5168 (males), IEBR A.5169 (female); Vietnam: Ha Giang Province: Bac Me Nature Reserve: IEBR A.5018 (male); Vietnam: Phu Tho Province: IEBR A.5019–5020 (females).

*Quasipaataoi* (*n* = 6): Vietnam: Kon Tum Province: Ngoc Linh Nature Reserve: IEBR A.4997–4999 (males), IEBR A. 5000 and IEBR A.5037–5038 (females).
